# Primary stroke prevention worldwide: translating evidence into action

**DOI:** 10.1016/S2468-2667(21)00230-9

**Published:** 2021-10-29

**Authors:** Mayowa O Owolabi, Amanda G Thrift, Ajay Mahal, Marie Ishida, Sheila Martins, Walter D Johnson, Jeyaraj Pandian, Foad Abd-Allah, Joseph Yaria, Hoang T Phan, Greg Roth, Seana L Gall, Richard Beare, Thanh G Phan, Robert Mikulik, Rufus O Akinyemi, Bo Norrving, Michael Brainin, Valery L Feigin, Stroke Experts Collaboration Group, Carlos Abanto, Semaw Ferede Abera, Adamu Addissie, Oluwadamilola Adebayo, Amos Olufemi Adeleye, Yerzhan Adilbekov, Bibigul Adilbekova, Thierry Armel Adoukonou, Diana Aguiar de Sousa, Temitope Ajagbe, Zauresh Akhmetzhanova, Albert Akpalu, Jhon Álvarez Ahlgren, Sebastián Ameriso, Silva Andonova, Foloruso Emmanuel Awoniyi, Moiz Bakhiet, Miguel Barboza, Hamidon Basri, Philip Bath, Olamide Bello, Dániel Bereczki, Simone Beretta, Aaron Berkowitz, Antonio Bernabé-Ortiz, Julie Bernhardt, Guna Berzina, Mher Bisharyan, Pascal Bovet, Hrvoje Budincevic, Dominique Cadilhac, Valeria Caso, Christopher Chen, Jerome Chin, Kamil Chwojnicki, Adriana Conforto, Vitor Tedim Cruz, Marco D'Amelio, Kristine Danielyan, Stephen Davis, Vida Demarin, Robert Dempsey, Martin Dichgans, Klara Dokova, Geoffrey Donnan, Mitchell S. Elkind, Matthias Endres, Urs Fischer, Fortuné Gankpé, Andrés Gaye Saavedra, Artyom Gil, Maurice Giroud, Elena Gnedovskaya, Vladimir Hachinski, Melanie Hafdi, Randah Hamadeh, T. Kolapo Hamzat, Graeme Hankey, Mirjam Heldner, Etedal Ahmed Ibrahim, Norlinah Mohamed Ibrahim, Manabu Inoue, Sungju Jee, Jiann-Shing Jeng, Yogesh Kalkonde, Saltanat Kamenova, Bartosz Karaszewski, Peter Kelly, Taskeen Khan, Stefan Kiechl, Aida Kondybayeva, Janika Kõrv, Michael Kravchenko, Rita V. Krishnamurthi, Jera Kruja, Mongkol Lakkhanaloet, Peter Langhorne, Pablo M. Lavados, Zhe Kang Law, Abisola Lawal, Maria Lazo-Porras, Dmytro Lebedynets, Tsong-Hai Lee, Thomas Leung, David S. Liebeskind, Patrice Lindsay, Patricio López-Jaramillo, Paulo Andrade Lotufo, Julia Machline-Carrion, Akintomiwa Makanjuola, Hugh Stephen Markus, Juan Manuel Marquez-Romero, Marco Medina, Sabina Medukhanova, Man Mohan Mehndiratta, Alexandr Merkin, Erkin Mirrakhimov, Stephanie Mohl, Miguel Moscoso-Porras, Annabel Müller-Stierlin, Sean Murphy, Kamarul Imran Musa, Ahmed Nasreldein, Raul Gomes Nogueira, Christian Nolte, Jean Jacques Noubiap, Nelson Novarro-Escudero, Yomi Ogun, Richard Ayobami Oguntoye, Mohammed Ibrahim Oraby, Morenike Osundina, Bruce Ovbiagele, Dilek Necioglu Orken, Atilla Özcan Ozdemir, Serefnur Ozturk, Melanie Paccot, Jurairat Phromjai, Piradov Piradov, Thomas Platz, Tatjana Potpara, Annemarei Ranta, Farooq Rathore, Edo Richard, Ralph L. Sacco, Ramesh Sahathevan, Irving Santos Carquín, Gustavo Saposnik, Fred Stephen Sarfo, Mike Sharma, Kevin Sheth, A. Shobhana, Nijasri Suwanwela, Irina Svyato, P.N. Sylaja, Xuanchen Tao, Kiran T. Thakur, Danilo Toni, Mehmet Akif Topcuoglu, Julio Torales, Amytis Towfighi, Thomas Clement Truelsen, Alexander Tsiskaridze, Marshall Tulloch-Reid, Nicolás Useche, Peter Vanacker, Sophia Vassilopoulou, Gorana Vukorepa, Vladimira Vuletic, Kolawole W. Wahab, Wenzhi Wang, Tissa Wijeratne, Charles Wolfe, Yared Mamushet Yifru, Adriana Yock-Corrales, Naohiro Yonemoto, Laetitia Yperzeele, Puhong Zhang

**Affiliations:** a Center for Genomic and Precision Medicine, College of Medicine, University of Ibadan, Ibadan, Nigeria; b Institute for Advanced Medical Research and Training, College of Medicine, University of Ibadan, Ibadan, Nigeria; c Stroke and Ageing Research, Department of Medicine, School of Clinical Sciences, Monash University, Melbourne, VIC, Australia; d Department of Neurology, Monash University, Melbourne, VIC, Australia; e Monash Health, and Peninsula Clinical School, Monash University, Melbourne, VIC, Australia; f Nossal Institute for Global Health, University of Melbourne, Melbourne, VIC, Australia; g Department of Neurology, Hospital de Clínicas de Porto Alegre, Porto Alegre, Brazil; h Department of Neurology, Universidade Federal do Rio Grande do Sul, Rio Grande do Sul, Brazil; i Department of Neurology, Hospital Moinhos de Vento, Porto Alegre, Brazil; j Brazilian Stroke Network, São Paulo, Brazil; k School of Public Health, Loma Linda University, Loma Linda, CA, USA; l School of Public Health, Christian Medical College, Ludhiana, Punjab, India; m Department of Neurology, Kasr Alainy School of Medicine, Cairo University, Cairo, Egypt; n Department of Neurology, University College Hospital, Ibadan, Nigeria; o Menzies Institute for Medical Research, University of Tasmania, Hobart, TAS, Australia; p Institute for Health Metrics Evaluation, University of Washington, Seattle, WA, USA; q Institute for Health Metrics Evaluation, University of Washington, Seattle, WA, USA; r Developmental Imaging Group, Murdoch Children's Research Institute, Melbourne, VIC, Australia; s International Clinical Research Center, Neurology Department, St Anne's University Hospital, Masaryk University, Brno, Czech Republic; t Department of Clinical Sciences, and Department of Neurology, Skåne University Hospital, Lund University, Lund, Sweden; u Department of Neuroscience and Preventive Medicine, Danube University Krems, Krems an der Donau, Austria; v National Institute for Stroke and Applied Neurosciences, School of Clinical Sciences, Faculty of Health and Environmental Sciences, Auckland University of Technology, Auckland, New Zealand; w Scientific and Educational Department, Research Centre of Neurology, Moscow, Russia

## Abstract

Stroke is the second leading cause of death and the third leading cause of disability worldwide and its burden is increasing rapidly in low-income and middle-income countries, many of which are unable to face the challenges it imposes. In this Health Policy paper on primary stroke prevention, we provide an overview of the current situation regarding primary prevention services, estimate the cost of stroke and stroke prevention, and identify deficiencies in existing guidelines and gaps in primary prevention. We also offer a set of pragmatic solutions for implementation of primary stroke prevention, with an emphasis on the role of governments and population-wide strategies, including task-shifting and sharing and health system re-engineering. Implementation of primary stroke prevention involves patients, health professionals, funders, policy makers, implementation partners, and the entire population along the life course.

## Introduction

The burden of stroke is a huge public health issue of growing importance. In 2019, stroke was the second leading cause of death (6·6 million people) and disability (143 million disability-adjusted life years lost [DALYs]) worldwide.^1^ During the past three decades, in absolute terms, global stroke incidence increased by 70%, its prevalence increased by 85%, its mortality increased by 43%, and DALYs due to stroke increased by 32%, with a greater increase in stroke burden in low-income and middle-income countries (LMICs) than in high-income countries (HICs).^1^ From 1990 to 2019, there was a 37% global increase in the total number of stroke-related DALYs due to risk factors, with LMICs disproportionately affected.^1^ Stroke-related DALYs attributable to risk factors increased by 48% in LMICs and declined by 25% in HICs between 1990 to 2019.^1^ In 2019, the five leading risk factors of stroke were high systolic blood pressure, high body-mass index, high fasting plasma glucose concentrations, ambient particulate matter (PM_2·5_) pollution, and smoking.^1^

In 2011, the UN resolution^2^ and the WHO Global NCD Action Plan 2013–20^3^ called on all governments to give primary prevention of non-communicable diseases (NCDs), including stroke, the highest priority of all diseases. The goal set by the UN resolution^2^ and the WHO Global NCD Action Plan 2013–20^3^ for countries was to achieve a 25% reduction in their NCD-related burden by 2025.^2^, ^3^ However, as stated by the UN Secretary-General in 2017,^4^ the current level of progress on the prevention and control of NCDs is insufficient to meet this goal.

The already enormous and continuously growing burden of stroke presents several challenges. Although LMICs bear most of the burden, they have only a small share of the global financial and health-care resources.^5^ Strokes also occur about 15 years earlier among individuals in LMICs than in HICs,^6^ leading to a marked negative impact on socioeconomic development as people in LMICs are most affected at the peak of their productive lives.^7^ Additionally, despite the currently available knowledge of evidence-based interventions for stroke prevention, this knowledge has not been translated into reduced stroke burden in LMICs due to barriers limiting implementation.^8^

The growing burden of stroke across the globe strongly suggests that current primary stroke and cardiovascular disease prevention strategies are either not used widely enough or are insufficiently effective. Comprehensive reviews of primary and secondary prevention of stroke^9^, ^10^ have mostly focused on individual risk factors and measurements of the effectiveness of preventive interventions. This Health Policy paper is based on a multifaceted approach, including a critical review of existing primary prevention strategies and current guidelines, economic analysis, and identification of gaps in primary stroke prevention. This approach enabled us to provide evidence-based pragmatic solutions on strategies for primary stroke prevention within a cost framework that global, regional, and national policy makers can use to reduce the burden of stroke across the globe, especially in LMICs. To derive these solutions (see appendix pp 21–22, we used four steps, with the first two steps described in detail and published elsewhere.^11^, ^12^ We conducted a situational evaluation by collecting and analysing data on the state of stroke-related services and resources in LMICs compared with HICs.^11^, ^12^ Priority setting was then done by extracting the highest grade of evidence-based recommendations, as identified from a systematic review of all available stroke guidelines across the globe.^12^ We derived the barriers and facilitators for implementing these recommendations in the context of LMICs from situational evaluation and relevant literature review (table 1 and table 2). Finally, we devised a roadmap for primary prevention and pragmatic solutions were proffered to implement evidence-based recommendations to reduce the burden of stroke in LMICs and other underserved settings.

**Table 1:** 2021–30 primary stroke prevention roadmap

	**Goals**	**Targets**	**Recommendations and actions**	**Assessment methods**
Scarcity of funding for primary stroke prevention across all countries, particularly in LMICs	To provide sufficient funding for primary and secondary stroke prevention	Governments and politicians	Encourage all governments and politicians to reinvest revenues from taxation on unhealthy products (eg, tobacco, sugary drinks, alcohol, and salt in processed foods, aimed at reducing consumption) back into health services and preventive strategies; all health-care policy makers should be aware that, for every US$1 spent on prevention of stroke and cardiovascular disease, there are over $10 returns on investment	The proportion of funding allocated to primary stroke prevention
Few countries or regions have established action plans for stroke prevention	To establish country-specific action plans and stroke prevention guidelines for every country	The whole population for population-wide prevention strategies and individuals at any level of risk for individual prevention strategies	All governments should allocate sufficient funding for the development and implementation of primary stroke prevention strategies, have financially sustainable action plans for primary and secondary stroke prevention, and should have culturally appropriate guidelines for primary and secondary stroke prevention; adults are encouraged to use freely available and validated mobile phone apps for managing their risk factors (eg, WSO, World Heart Federation, World Federation of Neurology, and European Stroke Organisation recommended Stroke Riskometer app); transferring and sharing tasks of primary stroke prevention from highly trained health professionals to less qualified health-care workers after training; culturally appropriate education about healthy lifestyles should be incorporated into standard education curricula and started early in life, with reinforcement across the lifespan	Stroke incidence, mortality, and disability; prevalence of risk factors; 5 or 10 year risk of cardiovascular disease and stroke; availability of stroke and transient ischaemic attack and stroke prevention clinics; proportion of people at risk of stroke and people who have had a stroke or transient ischaemic attack managed in clinics; proportion of evidence-based decisions in stroke prevention
Absence of an integrative approach in primary stroke prevention, particularly in LMICs	To establish collaboration between different national and international agencies and organisations involved in primary prevention of non-communicable disease	National and international agencies and organisations	Include nationally and internationally recognised stroke experts in all relevant national and international agencies and organisations involved in primary prevention of non-communicable diseases; prioritise primary stroke prevention strategies to reduce exposure to cardiovascular disease risk factors in the whole population across the life course, including intrauterine life, with a focus on optimal maternal and child health care, behavioural, and lifestyle risk factors, which would enable an integrative approach that also targets other non-communicable diseases (eg, dementia, diabetes, cancer, and pulmonary diseases)	Checklist of representation of stroke experts in all relevant national and international agencies and organisations involved in primary prevention of non-communicable diseases
Little stroke awareness across all countries	To establish national ongoing stroke awareness campaigns about stroke, its warning signs, and its prevention	The whole population	All national and regional stroke organisations should conduct ongoing stroke awareness campaigns about stroke, its warning signs, and its prevention, coordinated by the WSO; regular television programmes are the preferred channel of media for such campaigns	Stroke awareness surveys
Absence of monitoring systems for evaluation of the effectiveness of preventive strategies	To establish national and subnational (for large countries) monitoring frameworks	Whole population and people at risk of stroke	All countries should have monitoring systems to evaluate the effects of primary and secondary prevention strategies; in the absence of sufficient quality country-specific epidemiological data on burden of stroke and risk factors, health-care policy makers should be encouraged to use relevant Global Burden of Disease estimates; regular use of accurate data to support decision making	Changes in the 5 year or 10 year absolute risk of stroke and cardiovascular disease of outpatients; strengthening surveillance for key stroke risk factors (eg, increased blood pressure, smoking, alcohol, obesity, and excessive salt consumption) with use of regular (eg, once in 2–5 years) inexpensive population-based surveys (eg, WHO STEPwise survey) would provide policy makers with accurate estimates of prevalence of stroke risk factors to prioritise investments to reduce exposure to the risk factors and reduce the incidence and burden of stroke; ongoing or regular (eg, once in 2–5 years) registries of strokes morbidity and mortality
Insufficient funding of stroke prevention research across all countries, particularly in LMICs	To study determinants of stroke occurrence and outcomes and the best strategies to reduce stroke burden	Health research funding agencies	In consultation with recognised regional experts on stroke and public health, allocate sufficient funding for research in primary and secondary stroke prevention	Proportion of research funding allocated to primary stroke prevention (compared with the total health research funding)

LMICs=low-income and middle-income countries. WSO=World Stroke Organization.

**Table 2:** Evidence and pragmatic solutions for improving primary stroke prevention worldwide

	Level of evidence*	**Resources required for implementation**	**Ethical, legal, and social implications and barriers and facilitators**	**Recommendation for contextualisation and implementation through policy makers and other activities**
Countries should have government-endorsed policies for community-wide stroke prevention^3^, ^13^, ^14^, ^15^, ^16^, ^17^	Level B evidence that tobacco, salt, and alcohol taxation is an effective strategy to improve health; level A evidence for population-wide primary stroke and other non-communicable disease prevention	Expertise in stroke and cardiovascular disease epidemiology and public health	Industry lobbying (eg, for reducing salt content in processed food, and reducing consumption of sugary drinks and alcohol); absence of expertise to develop an efficient action plan and community support for introducing taxation on salt, sugary drinks, alcohol, and tobacco products; government and health policy engagement; public resources for accessible and affordable healthy food outlets, physical activity facilities, and healthy ecological environment	Policy makers and health experts to develop legislative changes for reducing salt content in processed food and reducing consumption of sugary drinks and alcohol, including the development of policies for community-wide stroke prevention activities, monitoring the effectiveness of prevention activities, and health-care workforce development; reinvestment of taxation revenue into primary and secondary prevention, health service development, and health research; health ministry order for public health services; developing and regularly (at least every 5 years) updating national primary stroke prevention guidelines; reinvestment of taxation revenue into the development of accessible and affordable healthy food outlets, physical activity facilities, and reducing air pollution
Countries should have ongoing stroke awareness and prevention campaigns and interventions^18^, ^19^, ^20^, ^21^, ^22^, ^23^, ^24^, ^25^, ^26^, ^27^	Level B evidence; WHO One Health initiative; level A evidence for control of risk factors for stroke prevention; level A evidence for use of polypill for blood pressure and cholesterol reduction	Expertise in development and maintenance of awareness campaigns; electronic patient management systems	Barriers include scarcity of engagement of stakeholders (eg, patients, providers, and policy makers); absence of collaboration between multiple sectors of society (eg, government, public health, and research and education)	Policy makers and health experts to develop strategies and action plans for ongoing stroke awareness and primary prevention, with a strong emphasis in LMICs on early detection and management of increased blood pressure and reduction of exposure to air pollution; policy makers and health experts should develop a plan for prioritising multisectoral and cost-effective accessible and affordable interventions, including the implementation of mobile technologies to promote a healthy lifestyle and primary stroke prevention; adequate education and regular antenatal care for pregnant women (balanced and adequate nutrition for pregnant women and infants are important primordial measures to reduce the risk of stroke)
Countries should have a nationwide and representative system for measuring and monitoring the effects of primary prevention activities (eg, absolute risk of stroke and cardiovascular disease of the population, stroke incidence, and stroke mortality)^27^	Level B evidence	Expertise in epidemiology, data management, and statistics to support ongoing monitoring of stroke	Barriers include absence of infrastructure to support a monitoring programme; expertise to develop an efficient programme; capacity to analyse the data collected and produce quality statistics; and data use to drive decision making	Policy makers and health experts to develop, implement, and monitor a reliable, simple, and fit-for-purpose strategic action plan with all stakeholders to ensure the availability of standardised surveillance systems for stroke and risk factors in their countries and regions

* Levels of evidence are randomised controlled trials (A), controlled trials with no randomisation (B), observational trials (C), and opinion of an expert panel (D).

In this Health Policy paper, we aimed to: provide an understanding of the burden and cost of stroke and the evidence for the cost-effectiveness of the existing primary stroke prevention strategies; provide an overview of available strategies, services, and guidelines for primary prevention of stroke and identify deficiencies and gaps in primary stroke prevention; and provide a set of pragmatic solutions for policy makers, funding organisations, and other stakeholders for funding and implementation of primary stroke prevention strategies, with examples of successful translation of evidence into actions. To achieve the aims of this paper, we structured the materials into six sections. First, we provide an overview of the cost of stroke and present the economic case for stroke prevention. Second, we provide an analysis of epidemiological evidence and situational analyses for improving primary prevention. Third, we provide an overview of barriers and facilitators for the implementation of primary stroke prevention guidelines and identify deficiencies, gaps, and pragmatic solutions in primary stroke prevention. Fourth, we describe joint efforts required for improving primary stroke prevention and justify the need for the establishment of regional and national plans on stroke prevention. Fifth, we present strategies for innovative dissemination and implementation of the suggested solutions. Finally, we define future directions in implementation and research on primary stroke prevention.

## The cost burden of stroke and the economic case for prevention

To develop an economic case for the pragmatic solutions for the primary prevention of stroke, we measured the global economic burden of stroke. The estimated economic burden accounted for the global financial costs of providing acute care to patients with stroke in hospital, including rehabilitation, and in the post-acute phase, including medical care, social services, and informal caregiving (appendix pp 7–20). We also estimated the income losses associated with people who have had a stroke and their households due to premature death and disability, and we estimated production losses as a result of stroke (appendix pp 7–20). Data from the Global Burden of Disease Study (GBD) 2017 on the number of new stroke cases and deaths from stroke^28^ were combined with the best available estimates of the costs of stroke treatment, rehabilitation, social care, and informal caregiving from 30 countries representing different income levels (eg, Germany, the USA, the UK, Japan, China, Brazil, Turkey, Cameroon, Thailand, and India). Our estimates suggest that the global lifetime costs of treatment, rehabilitation, social care, and informal caregiving are international (I) $393 billion (or 0·3% of global gross domestic product [GDP]), with a range from approximately $206 billion (0·2% of global GDP) to $638 billion (0·5% of global GDP) among new stroke cases in 2017. In addition to direct costs, income losses due to incident stroke cases from premature death and disability after stroke were likely to be large, which has important welfare implications for people who have had a stroke and their households. We estimated that discounted lifetime economic losses to households with incident stroke cases in 2017 were I$976 billion globally (0·77% of global GDP). Roughly a third of the income losses to households with incident stroke occurred in HICs and a half occurred in upper middle-income countries (MICs). Income losses from stroke in lower MICs and lower-income countries (LICs) accounted for only 15% of the global total, primarily because of their much lower income levels than HICs and upper MICs. However, these estimates are likely to be conservative because they do not account for out-of-pocket costs and income losses arising from the added responsibilities falling upon caregivers who might have to give up paid work. Using a friction cost approach, we estimated a lower bound of I$58 billion in production losses. Overall, we estimated that the total global direct and indirect costs of stroke in 2017 were US$891 ($746–1077) billion (1·12% [0·94–1·36%] of global GDP) or I$1369 ($1182–1614) billion (appendix p 15).

Although large, our estimates of the global economic burden of stroke cannot be directly compared with existing global economic burden estimates for other health conditions (eg, cancer,^29^ dementia,^30^ cardiovascular disease,^31^ and heart failure^32^) owing to differences in methods, especially relating to the issue of attribution of costs to conditions from similar risk factors. A World Economic Forum report in 2011 did provide comparable estimates for the economic burden of different NCDs, but did not include stroke.^33^ Given the average annual growth rate of the global economy of approximately 3·5% during the past decade, our estimates of the economic burden of stroke translates to at least 10% of the annual addition to global wealth being devoted to stroke. This economic burden, in addition to the economic consequences to households directly affected by stroke, shows major declines in the wellbeing of people who have had a stroke and their households.

With the costs of stroke care, the economic gains from interventions that can help reduce stroke incidence and mortality by small numbers are potentially large. There is a sufficient body of evidence to show that achieving the UN Sustainable Development Goals (SDGs;^34^ appendix pp 20–21) and WHO health targets^3^ with low costs (eg <US$1 per person a day [$0·43–0·90] in LICs and <$3 a day [$0·54–2·93] in MICs)^35^, ^36^ could reduce the mortality rate for ischaemic heart disease and stroke by 10%. In turn, economic losses in LMICs would be reduced by an estimated US$25 billion per year.^36^, ^37^ Another promising strategy to reduce stroke incidence and mortality is to reprioritise health investment streams towards population-wide primary prevention across the lifespan. It has been estimated that, for every US$1 spent on the prevention of stroke and cardiovascular disease, there is a more than $10 return on investment and that the preventive interventions focused on risk factors are the most cost-effective options.^38^ Additionally, stroke primary prevention efforts are likely to yield large gains due to secondary effects of reducing the risk of heart disease, type 2 diabetes, dementia, and some types of cancer that share common risk factors, thus supporting achievements of a range of SDGs.

## Epidemiological evidence and situational analyses for improving primary prevention

A World Stroke Organization coordinated international survey on stroke^11^, ^12^ showed that only about a third of the recommended activities for primary prevention are being done in the 82 countries that participated in the survey, and the use of these recommended activities was particularly poor in LICs. Although 81% of countries reported that cardiovascular disease risk stratification was offered at primary health-care facilities, the reported availability of this service within these countries varied widely. Although nearly half (38 of 82) of these countries reported that risk stratification was available in more than 50% of health-care facilities, 29% reported that it was available in less than 25% of facilities, and 15% reported that it was available in between 25% and 50% of facilities. Additionally, half of the countries (53%) reported general availability of all six essential tests and procedures (ie, measurement of height, weight, blood pressure, blood glucose, and total cholesterol, and urine strips for albumin assay).

Marked disparities were evident across the income groups: 96% of HICs reported all six tests and procedures were generally available compared with 16% of LICs. Although many countries have a national strategy towards a healthy diet, reducing tobacco use, and reducing diabetes, only 42% of countries have national strategies for all three issues and fewer than one in three countries have smoke-free environments in all indoor workplaces, public transport, and indoor public places.

There are two main primary stroke and cardiovascular disease prevention strategies currently in use: population-wide cardiovascular disease strategies and strategies for individuals who have high risk for cardiovascular disease. Conventional screening of the population for people with high risk of cardiovascular disease risk with various prediction algorithms (eg, atherosclerotic cardiovascular disease risk evaluation^39^ or PREDICT algorithms^40^), which categorise people into mild (ie, moderate), low, or high cardiovascular disease risk, does not motivate people with low and mild cardiovascular disease risk to reduce their risk.^41^ Therefore, the World Stroke Organization and World Heart Federation, in their recent joint position statement,^42^ recommended not to label people with low and mild cardiovascular disease risk when communicating absolute cardiovascular disease risk to patients. These screenings of the population for high cardiovascular disease risk have been shown to be ineffective to reduce stroke and ischaemic heart disease incidence and mortality rates in randomised trials in 107 421 people (relative risk 1·05 [95% CI 0·95–1·17]; 53%).^18^, ^43^, ^44^ Because of the need for a blood lipid test, and the associated costs, these prediction tools have low applicability in LMIC settings (until point-of-care devices with low cost are available).^41^ Moreover, as stated by the World Heart Federation and World Stroke Organization,^42^ these screening programmes might exacerbate socioeconomic inequalities^45^ and have potential hazards of labelling people as low risk, giving them false reassurance that they are protected from stroke and heart attack and compromising any motivation to control risk factors.^41^, ^45^, ^46^ So-called high-risk prevention strategies are targeted interventions for individuals at high risk of cardiovascular disease that are usually implemented by health-care professionals. Although these interventions might be adequate for conditions confined to an identifiable minority of people at high risk of cardiovascular disease, most (up to 80%) of stroke and cardiovascular disease arise from individuals at low risk,^47^, ^48^, ^49^ who not covered by the high-risk prevention strategies.^41^ Issues for clinicians in primary stroke prevention at the individual level include the absence of digital decision-making tools^19^ and scarcity of time to motivate, develop, and give tailored primary prevention recommendations to the patient. An example of a digital decision-making tool that can help to resolve all these issues is the desk-top multilanguage PreventS web app for clinicians, developed by the Auckland University of Technology (appendix pp 22–27).

**Figure 1:**
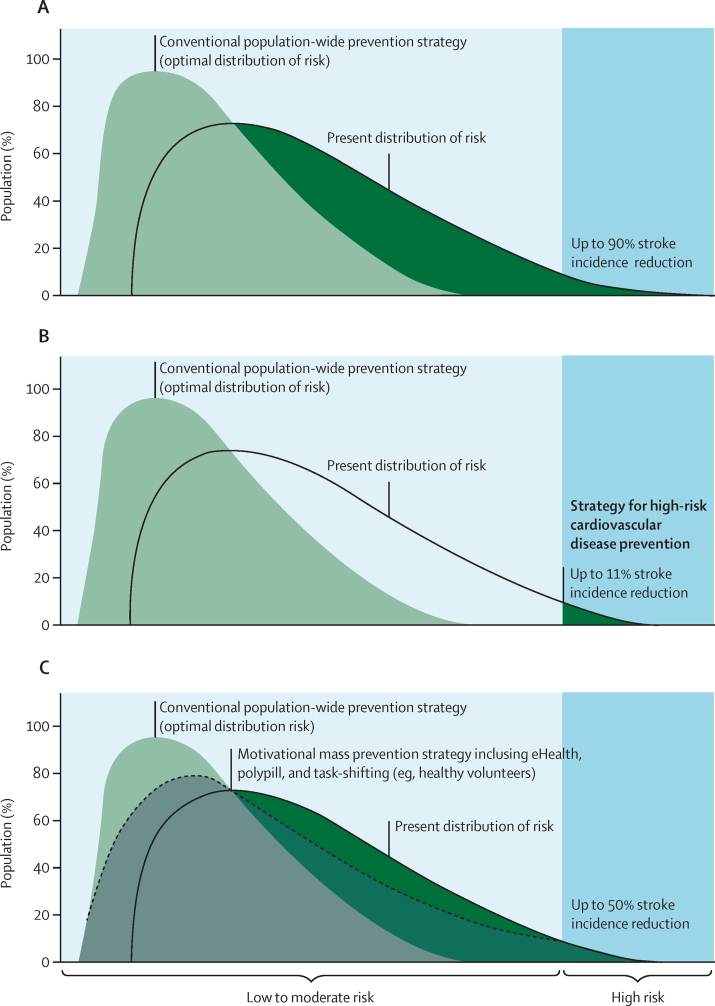
Optimal shift in the distribution of cardiovascular disease risks through a combination of population-wide strategies for high-risk cardiovascular disease prevention Areas shadowed in green show a theoretically possible proportion of the population that could benefit from (A) a population-wide prevention strategy, (B) strategies for high-risk cardiovascular disease prevention, and (C) a motivational mass individual risk prevention strategy regardless of the cardiovascular disease risk (ie, use of mobile applications to reduce lifestyle and other risk factors). Modified from Feigin and colleagues, with permission.^52^

As the mean amount of exposure to causal risk factors throughout the population correlates closely with the incidence of stroke and cardiovascular disease in the population, the population-based strategy of prevention aims to reduce the mean amount, and overall distribution, of exposure to causal risk factors throughout the population to reduce the incidence of cardiovascular disease.^50^ Preliminary calculations suggest that, if population-wide strategies were implemented widely and effectively, they could prevent up to 50–90% of stroke and cardiovascular disease events in 5 years.^1^, ^20^, ^46^, ^51^ A motivational mass prevention strategy via eHealth technologies^52^, ^53^ in combination with polypill and task-shifting, task-sharing (or task transfer)^46^ could prevent up to 50% of stroke and cardiovascular disease events. High-risk strategies can potentially prevent about 11% of stroke and cardiovascular disease events (figure 1).^54^ Both high-risk and population-wide strategies should complement each other,^21^, ^22^ and priority should be given to population-wide strategies.^41^, ^42^

## Guidelines and pragmatic solutions

Population-wide strategies for primary stroke and cardiovascular disease prevention are well established (eg, nationwide measures to reduce exposure to smoking and vaping; reduce intake of sugary drinks, salt, and alcohol; and promote adequate physical activity).^3^ These population-wide strategies are recommended in several international and WHO guidelines,^35^, ^55^, ^56^, ^57^ but their implementation is unacceptably slow and far from universal.^12^ As shown in a systematic review of stroke guidelines,^12^ there are two main reasons for slow implementation of population-wide strategies. First, such strategies require policy and legislative changes that are often not supported by major industries (eg, salt reduction in processed food and reduction of exposure to smoking, alcohol, and fast food).^23^, ^58^ According to the GBD 2019 estimates, poor and unhealthy diet, as defined by a cluster of dietary risks, is associated with 30·5% (95% uncertainty intervals 22·6–39·8) of stroke-related DALYs.^1^ There is also evidence of the double burden of low income and an unhealthy neighbourhood food environment on objective measures of adiposity and its contribution to social inequalities in health.^24^ Governments should be responsible for, and seek strategies to reduce the exposure of their citizens to, unhealthy food, and should facilitate healthy diets and healthy fast food chains as the distributors of snacks, dinners, and lunches. Second, implementation of a full range of population-wide prevention strategies requires substantial investments from governments and industry, and preferably universal health coverage, including setting up affordable and widely accessible health services, creating affordable facilities for physical activity, and reducing air pollution and socioeconomic inequalities. In addition, despite a special 2011 NCDs UN declaration to have an NCD prevention plan in every country,^2^ most countries still do not have such a plan.

The majority of the burden of stroke (60–70%) across all countries in the world is associated with elevated systolic blood pressure (the single most important risk factor for stroke) and unhealthy lifestyle risk factors, such as smoking, obesity, low physical activity, and poor diet (eg, excessive salt, sugar, and alcohol intake and low fruit and vegetable consumption).^1^ A comprehensive review of hypertension prevalence and treatment found that, although the overall rate of hypertension in the world did not change much from 1990 to 2019, the burden of hypertension has shifted from HICs to LMICs and the number of people with hypertension has doubled from 648 million in 1990 to 1·28 million since 1990.^59^ The new WHO *Guideline for the pharmacological treatment of hypertension in adults*^60^ provides recommendations on the threshold for the initiation of pharmacological treatment for hypertension (systolic blood pressure of ≥140 mm Hg or diastolic blood pressure of ≥90 mm Hg in people with a confirmed diagnosis of hypertension or systolic blood pressure of 130–139 mm Hg in people with existing cardiovascular disease, regardless of the absolute cardiovascular disease risk) and recommendations on intervals for follow-up, target blood pressure for control, and the skill level of health-care workers who can initiate treatment. Reducing exposure to these risk factors and treating hypertension should be the priority targets for both population-wide and individual-based preventive interventions for primary stroke prevention (panel). A good example of decisive actions to stop smoking is the suite of proposals of the Government of New Zealand aimed at creating a smoke-free generation and moving the country closer to its goal of being smoke free by 2025. Step-by-step action plans and online courses on global salt reduction strategies for policy makers, advocates, and programme managers have been developed to implement scalable interventions focusing mainly on LMICs and are offered for free by Johns Hopkins University. The medical community must continue to advocate for governments to implement evidence-based population-wide prevention strategies.

Panel **Key solutions for primary stroke prevention**

Effective stroke prevention should include population-wide and individual-based strategies that cover all, or most, of the population, with priority given to population-wide strategies.

Individual-based primary stroke prevention strategies can be best accomplished with mobile phone technology (so-called motivational mass individual strategy for stroke prevention),^41^ a simple and inexpensive screening for a history of cardiovascular disease and presence of modifiable risk factors (particularly smoking and vaping, obesity, and elevated blood pressure), linked to local, regional, or national electronic databases for health care. These strategies for the individuals should be enhanced by the involvement of health professionals who use similar motivational computer-based tools to individualise stroke risk factor recommendations and monitor the effects of the interventions among individuals. Individual-based strategies can also be accomplished with shifting and sharing of tasks from highly trained health professionals to health-care workers, particularly community-based health workers to facilitate stroke prevention interventions on the individual. Examples of individual-based primary stroke prevention strategies are those in selected populations of Finland,^61^ Japan,^62^ and the USA;^63^ the validated and free Stroke Riskometer app^52^, ^53^, ^64^ that is being used in 19 languages in 78 countries, potentially covering 5·3 billion people; the PreventS-MD web app for clinicians;^1^ and the transferring and sharing of tasks from highly trained health professionals to health-care workers implemented in several areas of India.^65^, ^66^

Governments should provide adequate health services, improve socioeconomic conditions, reduce inequities, and influence environmental (eg, air pollution) and lifestyle factors (eg, smoking, vaping, reducing salt, sugar in processed foods, and alcohol intake through legislation and taxation), and health systems should identify, screen, and manage risk factors. Revenues from these taxations should be invested into the public health sector and health research to improve the health of the taxpayers, including appropriate funding of primary prevention strategies for stroke, cardiovascular disease, and other non-communicable diseases. Governments have to be transparent about the proportion of health budgets that are focused on prevention. Examples of population-wide strategies include effective smoking cessation campaigns in some countries;^13^ taxation of sugary drinks in several countries, including the UK, Ireland, France, Canada, South Africa, United Arab Emirates, Portugal, Mexico, and Sri Lanka;^14^ junk food taxes in Mexico and Hungary;^15^ successful alcohol reduction in Russia;^16^ and a successful air pollution campaign in China.^17^ The effectiveness of the proposed primary stroke prevention measures should be regularly assessed by monitoring stroke incidence, mortality, prevalence (ie, rates and absolute numbers), and risk factors (ie, prevalence and changes in absolute and relative risks of stroke and cardiovascular disease) at the individual and population levels. Examples of such monitoring include the World Stroke Organization stroke survey,^12^ WHO health survey,^26^ and the Global Burden of Disease Study.^27^

On the basis of the totality of evidence the World Stroke Organization has issued a Declaration^46^ that recommends the use of four strategies for global primary prevention of stroke and dementia: (1) population-wide policy strategies to reduce exposure to risk factors (including environmental risk factors such as air pollution) for stroke, dementia, cardiovascular disease, and other NCDs across the lifespan of the entire population regardless of the individual cardiovascular disease risk; (2) motivational population-wide strategy that uses health apps (eg, the free Stroke Riskometer app^44^, ^53^, ^52^, ^64^) or apps to reduce lifestyle and other risk factors in adults at any increased risk of stroke (appendix pp 23–27); (3) targeted polypill (consisting of two low-dose generic blood pressure-lowering medications and one generic lipid-lowering medication) strategy for adults older than 55 years at risk of cardiovascular disease (ie, at least two behavioural or metabolic cardiovascular disease risk factors); and (4) preventive strategies to control behavioural risk factors (especially smoking and elevated blood pressure) and diabetes via community health workers (community health workers were also suggested to facilitate implementation of the second and third strategies).

As stated in the World Stroke Organization Declaration,^46^ policy makers and health providers should reduce exposure to risk factors at a population level regardless of the cardiovascular disease risk through mass approaches (eg, smoking cessation campaigns, reducing salt and sugar in processed food, and restricting alcohol consumption) and more individual-focused motivational education about behavioural risks (eg, poor diet, physical inactivity, alcohol use, and smoking) via apps. In addition, simple inexpensive screening for vascular risks (eg, elevated blood pressure, smoking, and overweight or obesity)^44^ by community health-care workers or people from stroke support organisations (in resource-poor settings) or by medical professionals (including use of blood lipid tests; in settings with better resources) would identify individuals in need of prophylactic drug therapy in conjunction with lifestyle and behavioural interventions.^41^ There is also evidence of sex differences in the risk of stroke^1^ and its risk factors^67^ and evidence that the intensity of primary stroke prevention should not be reduced as people age.^68^ These recommendations are summarised in the 2021–30 primary stroke prevention roadmap (table 1). With all these recommendations implemented into practice, a similar shift to that shown in figure 1 in the distribution of risk factors would occur as with the population-wide primary prevention strategy (figure 1).

## Joint efforts and establishment of regional and national plans

**Figure 2:**
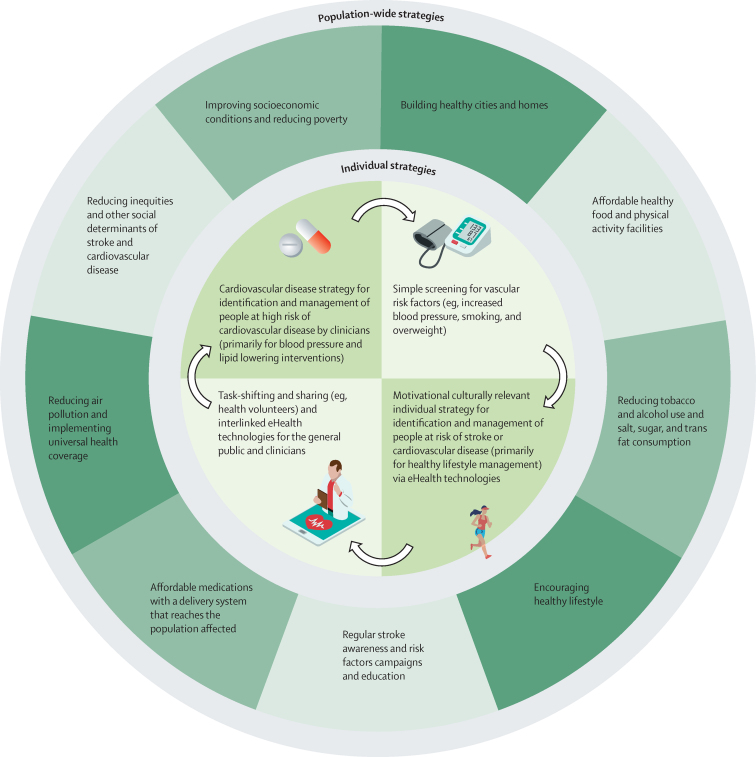
Action plan for governments and other policy makers for primary stroke prevention measures at the population (ie, socioeconomic, environmental, and behavioural) and individual levels

Stroke is a complex medical and socioeconomic issue.^25^, ^69^, ^70^ Therefore, the importance of global, international, and national efforts and collaboration between various sectors of health care, decision makers, government and non-government agencies (eg, stroke, cardiovascular disease, and NCD organisations), industry, communities, and individuals for effective reduction of stroke burden cannot be overemphasised (figure 2).^9^, ^71^ Government bodies have the power and responsibility to provide adequate health services to cover primary prevention, improve socioeconomic conditions, reduce inequities, and influence environmental (eg, reduction of air pollution and building healthy cities) and lifestyle factors (eg, reducing salt and sugar in processed food and alcohol intake through legislation and taxation). Moreover, health systems have responsibilities for identification and management of risk factors and people with cerebrovascular diseases, and government and non-government organisations have responsibilities for ongoing public (eg, stroke awareness days) and professional (eg, teaching courses and conferences) education. In addition, intersectoral intervention is required to provide essential medicines for primary stroke prevention (eg, affordable blood pressure-lowering and lipid-lowering medications) and an enabling environment for healthy lifestyles, including reworking the food chain to make healthy food available and affordable for all, providing safe neighbourhoods conducive to walking, and ensuring access to care. Another approach would be to change public policy to enable community health workers to distribute medicines prescribed by doctors. This approach is particularly important in hard-to-reach regions where there is little access to medical professionals. This type of coordinated intervention allows interlinking community-wide prevention and individual management approaches that improve health across the care continuum and across settings and strategies (figure 2).^72^

The development and implementation of action plans for primary stroke prevention should be aimed towards achieving the internationally recommended goals and targets for reducing the burden from NCDs.^2^, ^3^ These country-specific and financially sustainable action plans and consensus statements need to be developed by recognised local experts, based on evidence, and endorsed by government agencies and should contain well developed implementation plans that include key performance indicators, steps, timelines, funding (including funding for implementation), and accountable people. These action plans should be facilitated by national, culturally appropriate, and up-to-date guidelines for primary stroke prevention. Unfortunately, there is a shortage of operational national plans aligned with the Global Action Plan for NCDs.^73^ Although there are several national guidelines for primary stroke prevention in HICs,^55^, ^74^ there is a paucity of such evidence-based, context-appropriate pragmatic guidelines in LMICs.^9^

Although mainstream preventive strategies should be similar in HICs and LMICs, differences in the population-attributable risks, lifetime risk of stroke, distribution of different risk factors, and availability of resources should be considered when setting goals and priorities. For example, given the much greater burden of smoking, air pollution, and haemorrhagic stroke in LMICs than in HICs,^75^ a strong emphasis on early detection and management of elevated blood pressure, reduction of air pollution, and anti-smoking campaigns should be a priority in LMICs. This priority should be facilitated by government-imposed measures to reduce sodium in processed food and increase education of individuals about reducing salt and tobacco intake.^76^ In addition, in HICs, where smoking prevalence has reduced and the burden associated with ischaemic stroke is noticeably higher than in LMICs, it seems reasonable to focus more heavily on the reduction of other behavioural risks (particularly on the reduction of sugar consumption and physical inactivity) and on the identification and pharmacological or surgical management of medical conditions that lead to stroke, including hypertension, diabetes, and atrial fibrillation. Population-wide and individual primary stroke and cardiovascular disease prevention strategies,^52^ should be used regardless of the level of stroke or cardiovascular disease risk, with priority given to population-wide strategies. In addition to preventive strategies towards medical risk factors, addressing social determinants of health (eg, poverty, education, health insurance, social inclusion and non-discrimination, daily living conditions, and access to quality health services that are affordable) is of crucial importance for effective primary stroke prevention.^77^, ^78^ Addressing social determinants of health requires the involvement of a wide range of stakeholders both within and beyond the health sector and at all levels of government (figure 2).^2^, ^77^

Actions to improve stroke prevention come at a cost. With already overstretched health budgets, even in HICs, sustainable funding is an issue**.** One of the most promising strategies to secure such funding is to reinvest revenues from taxation on unhealthy products (eg, tobacco, sugary drinks, alcohol, and salt in processed food)^38^, ^78^, ^79^, ^80^, ^81^, ^82^, ^83^, ^84^ and the money saved from preventing stroke back into health services and preventive strategies.^72^ This strategy is important as reduced consumption of these unhealthy foods has been shown to be beneficial for stroke, cardiovascular disease, and overall health at the population level. Although it is widely acknowledged that prevention is better than cure, even HICs allocate less than 2–3% on average of their health spending to public health and prevention activities,^85^ and there is also evidence of substantial underfunding of stroke-related research.^86^ Governments have a leading role in the implementation of proven effective primary stroke prevention strategies and should be transparent about the proportion of health budgets that are focused on prevention.

Politicians and policy makers should realise that, without urgent improvement in the primary prevention of stroke and other major NCDs, the sustainability of the whole health system could soon be in question. Only by combining with other interventions for NCD prevention will stroke prevention inventions be fully impactful.^71^ The Global Alliance for Chronic Diseases is a good example of such an integrative approach. Several reports show the effectiveness of population-wide primary prevention strategies in selected populations of Finland,^61^ Japan,^62^ and the USA.^63^

## Innovative dissemination for substantial implementation and impact

The establishment of the global Stroke Control, Observatory, and Reduction Ecosystem (gSCORE; appendix p 25)^11^, ^87^ will be key to engage all stakeholders in society to: address key environmental factors via policy change (eg, the social determinants of health and making default choices healthy); enhance stroke awareness through key community influencers who can deliver culturally tailored messages with strategies, such as using social media (ie, social media influencers) and the arts (ie, music, comedy, film, and television); address motivation, self-efficacy, and self-management skills; and empower the stroke commissioners to be the champions and advocates of ensuring rigorous implementation and evaluation across the globe.

The gSCORE, using the WHO Global Action Plan for NCDs, is planned to operate at country, regional, and global levels in collaboration with relevant policy makers and implementation partners, including national and regional stroke, neurology, cardiovascular disease, and NCD organisations and relevant alliances.

## Conclusions and future directions

This Health Policy paper provides estimates of the global cost of stroke and evidence-based pragmatic solutions on strategies for primary stroke prevention, especially in LMICs. The proffered key solutions are targeted at reducing the occurrence of stroke and preventing economic losses from stroke through primary prevention across the life course. As many lifestyle habits are set early in life, culturally appropriate education about healthy lifestyles should be incorporated into standard education curricula, started early in life with reinforcement across the lifespan, and incorporate families. These preventive strategies should be complemented by adequate stroke education campaigns that consider cultural and subcultural differences, ethnicities, beliefs, geographical differences, and risk of stroke across the lifetime.

For an effective effort, there is a need for synergy between health-care providers, government and non-government agencies, industry, academic organisations, opinion leaders in society, and individuals. An approach that integrates strategies aimed at primary stroke prevention (population-wide and targeted strategies towards individuals with any level of increased stroke risk) with strategies aimed at prevention of other NCDs is most likely to be successful as many risk factors are shared between stroke and other NCDs. Establishing a global chronic disease prevention initiative with stroke as a major focus has also been suggested.^88^

As many stroke risk factors are common to other major NCDs, such as ischaemic heart disease, type 2 diabetes, renal disease, dementia, and some types of cancer, it is expected that the worldwide implementation of the solutions will not only halve the burden of stroke but also substantially reduce the burden from other major NCDs. This co-benefit would not only save millions of lives around the globe but could also have a substantial economic impact. The development of primary stroke prevention guidelines for LMICs is urgently required. The audience for future primary stroke prevention guidelines in HICs and LMICs should be broadened since many primary stroke prevention interventions require intersectoral funding, policy initiatives, and buy-in from the population. Additional research is required to develop integrative, culturally appropriate, and population-specific eHealth technologies for effective primary stroke prevention, including digital decision-making tools for clinicians and community health workers, and to establish the best balance between various primary stroke prevention strategies to maximise cost-effectiveness and minimise inequalities.

## Search strategy and selection criteria

We searched MEDLINE, Embase, Google Scholar, Google, and the Cochrane Library with the keywords in the title or abstract: “stroke”, “cerebrovascular disease”, “isch(a)emic stroke”, “intracerebral h(a)emorrhage”, “subarachnoid h(a)emorrhage”, “transient isch(a)emic attack”, or “cardiovascular disease” AND “prevention”, “cost”, “guidelines”, “awareness”, “tax or taxation”, “trial”, “policy”, “legislation”, “mHealth”, “eHealth”, “polypill”, “roadmap”, “incidence”, “prevalence”, “mortality”, “burden”, or “outcomes” for research published between Jan 1, 1980, and May 15, 2021, in English. We also searched websites of medical societies and stroke experts for additional stroke prevention guidelines. We focused our searches on population-based studies and guidelines related to primary stroke prevention published since Jan 1, 2011. Additionally, we manually searched the reference lists of relevant publications and consulted with experts in stroke, cardiovascular disease, and other relevant stakeholders to complement the electronic searches.

## Declaration of interests

VLF declares that the PreventS web app and Stroke Riskometer app are owned and copyrighted by Auckland University of Technology; has received grants from the Brain Research New Zealand Centre of Research Excellence (16/STH/36), Australian National Health and Medical Research Council (NHMRC; APP1182071), and World Stroke Organization (WSO); is an executive committee member of WSO, honorary medical director of Stroke Central New Zealand, and CEO of New Zealand Stroke Education charitable Trust. AGT declares funding from NHMRC (GNT1042600, GNT1122455, GNT1171966, GNT1143155, and GNT1182017), Stroke Foundation Australia (SG1807), and Heart Foundation Australia (VG102282); and board membership of the Stroke Foundation (Australia). SLG is funded by the National Health Foundation of Australia (Future Leader Fellowship 102061) and NHMRC (GNT1182071, GNT1143155, and GNT1128373). RM is supported by the Implementation Research Network in Stroke Care Quality of the European Cooperation in Science and Technology (project CA18118) and by the IRIS-TEPUS project from the inter-excellence inter-cost programme of the Ministry of Education, Youth and Sports of the Czech Republic (project LTC20051). BN declares receiving fees for data management committee work for SOCRATES and THALES trials for AstraZeneca and fees for data management committee work for NAVIGATE-ESUS trial from Bayer. All other authors declare no competing interests.
