# Correction to *Lancet Public Health* 2022; 7: e74–85

**DOI:** 10.1016/S2468-2667(21)00281-4

**Published:** 2022-01-04

*Owolabi MO, Thrift AG, Mahal A, et al. Primary stroke prevention worldwide: translating evidence into action.* Lancet Public Health *2022; **7:** e74–85*—In this Health Policy paper, estimates for discounted lifetime economic losses to households with incident stroke cases in 2017 should have been I$976 billion globally (0·77% of global GDP); the proportion of global income losses from stroke in lower middle-income countries and lower-income countries should have been 15%; the proportion of the income losses that occurred in households in high-income countries should have been roughly a third and a half in households from upper middle-income countries; and global direct and indirect costs of stroke in 2017 should have been US$891 ($746–1077) billion (1·12% [0·94–1·36%] of global GDP) or I$1369 ($1182–1614) billion, and the appendix has been corrected. The Stroke Experts Collaboration Group list has also been corrected. This correction has been made as of Dec 28, 2021.

