# Supplementary material for: Primary stroke prevention worldwide: translating evidence into action
Source: Lancet Public Health. 2021 Oct 29;7(1):e74–85. doi: 10.1016/S2468-2667(21)00230-9 (PMC8727355; doi:10.1016/S2468-2667(21)00230-9)
Supplement: Supplementary appendix [file mmc1.pdf]

THE LANCET
Public Health

Supplementary appendix

This appendix formed part of the original submission and has been peer reviewed. We post it as supplied by the authors.

This online publication has been corrected. The corrected version first appeared at thelancet.com/public-health on December 28, 2021

Supplement to: Owolabi MO, Thrift AG, Mahal A, et al. Primary stroke prevention worldwide: translating evidence into action. *Lancet Public Health* 2021; published online Oct 28. http://dx.doi.org/10.1016/S2468-2667(21)00230-9.

SUPPLEMENTARY MATERIALS

SECTION 1. STROKE EXPERT COLLABORATION GROUP 2
SECTION 2. STROKE COST ESTIMATES 7
METHODOLOGY OF ECONOMIC ANALYSIS 7
Estimating Direct Medical Costs of Stroke 7
Estimation of Income Losses 15
Estimation of Production Losses: Friction Cost Approach 18
SUMMARY 20
SECTION 3. PRIMARY STROKE PREVENTION and SDGs 21
SECTION 4. TANGIBLE SOLUTIONS READY FOR GLOBAL IMPLEMENTATION 21
eHealth technologies 23
SECTION 5. GLOBAL STROKE CONTROL OBSERVATORY AND BURDEN REDUCTION ECOSYSTEM (G-SCORE) 25
SECTION 6. FIGURES 26
REFERENCES 28

SECTION 1. STROKE EXPERT COLLABORATION GROUP

***Stroke Experts Collaboration Group (in alphabetical order, by last name)**
Abanto C. MD PhD[1], Abera S.F. MSc[2], Addissie A. MD MPH MA PhD[3], Adebayo O.I. MBBS[4], Adeleye A.O. MD[4], Adilbekov Y. MD, PhDr[5], Adilbekova B. PhD[6], Adoukonou T.A. MD[7], Aguiar de Sousa D. MD, PhD[8], Ajagbe, T. MBBS[4], Akhmetzhanova Z. MD, MSc[6], Akpalu A. MB, ChB[9], Álvarez Ahlgren J.H. MPH[10], Ameriso S.F. MD[11], Andonova S. MD, PhD, DSc[12], Awoniyi F.E. MSc[13], Bakhiet M. MD, PhD[14], Barboza, M.A. MD, MSc[15], Basri H. MD[16], Bath, P.M. DSc, FMedSci[17], Bello, O. MBBS[4], Bereczki D. MD, PhD, DSc[18], Beretta S. MD, PhD[19], Berkowitz A.L. MD, PhD[20], Bernabé-Ortiz, A. MD, PhD, MPH[21], Bernhardt J. PhD[22], Berzina G. MD, PhD[23], Bisharyan M.S. MD, DSc[24], Bovet P. MD, MPH[25], Budincevic H. MD, PhD[26], Cadilhac D A. PhD[27], Caso V. MD, PhD[28], Chen C. MD[29], Chin J.H. MD[30], Chwojnicki K. MD[31], Conforto A.B. MD[32], Cruz V.T. MD, PhD[33], D'Amelio M. MD[34], Danielyan K.E. PhD[35], Davis S. MD[36], Demarin V. MD, PhD[37], Dempsey R.J. MD[38], Dichgans M. MD[39], Dokova K. MD, PhD[12], Donnan G. MBBS, MD[40], Elkind M.S. MD, MS[41], Endres M. MD[42], Etedal I. MD[43], Fischer U. MD[44], Gankpé F. MD, MPH[45], Gaye-Saavedra A. MD[46], Gil, A. MD, PhD, MPH[47], Giroud M. MD[48], Gnedovskaya, E.V. MD, MSc[49], Hachinski V. CM, MD, DSc[50], Hafdi M. MD/Msc[51], Hamadeh R.R. DPhil[52], Hamzat T.K. PhD[53], Hankey G.J. MBBS, MD[54], Heldner M.R. MD, MSc[44], Ibrahim N.M. MBBCh[55], Inoue M. MD, PhD[56], Jee S. MD, PhD[57], Jeng J.S. MD, PhD[58], Kalkonde Y. MD, MSc[59], Kamenova S. MD[60], Karaszewski, B. MD[31], Kelly P. MD MS[61], Khan T. MD[62], Kiechl S. MD[63], Kondybayeva A. MD, PhD[60], Kõrv J. MD, PhD[64], Kravchenko M. MD, PhD[49], Krishnamurthi R.V. BSc MApplSc, PhD[65], Kruja J. MD, PhD[66], Lakkhanaloet M. MD[67], Langhorne P. MD, PhD[68], Lavados P.M. MD, MPH[69], Law Z.K. MD, PhD[55], Lawal A. MBBS[4], Lazo-Porras M. MD, MSc[21], Lebedynets D. MD[70], Lee T.H., MD, PhD[71], Leung T.W. MBChB[72], Liebeskind D.S. MD[73], Lindsay M.P. PhD[74], López-Jaramillo P. MD, PhD[75], Lotufo P.A. MD, DPH[76], Machline-Carrion M.J. MD, PhD[77], Makanjuola A. MBBS, MS[4], Markus H.S. MD, DM, F Med Sci[78], Marquez-Romero J.M. MD, MS[79], Medina M.T. PhD[80], Medukhanova S. MD, MPH[5], Mehndiratta Man Mohan MD, DM[81], Merkin A. MD, PhD[65] , Mirrakhimov E. MD[82], Mohl S.M. BA[83], Moscoso-Porras M. MSc[21], Müller-Stierlin A.S. PhD[84], Murphy S. MD[85], Musa K.I. PhD[86], Nasreldein A. MD[87], Nogueira R. MD[88], Nolte C.H. MD[89], Noubiap J.J. MD, MMed[90], Novarro-Escudero N. MD, MSc[91], Ogun Y. BSc, MBChB, MPH[92], Oguntoye A. MD[4], Oraby M.I. MD[93], Osundina M.A. MBBS[4], Ovbiagele B. MD, MAS[94], Ōrken D.N. MD, PhD[95], Ōzdemir A.O. PhD[96], Ozturk S. MD[97], Paccot M. MSc[98], Phromjai J. PhD[99], Piradov M. MD, PhD, D.Med.Sci[49], Platz T. MD[100], Potpara T. MD, PhD[101], Ranta A. MD, PhD[102], Rathore F.A. MBBS, MSc[103], Richard E. MD, PhD[104], Sacco R.L. MD, MS[105], Sahathevan R. MD, PhD[106], Santos C. IR. MD, MHA (C)[107], Saposnik G. MD, MSc, PhD[108], Sarfo F.S. MD, PhD[109], Sharma M. PhD[110], Sheth K.N. MD, PhD[111], Shobhana A. MD[112], Suwanwela N. C. PhD[113], Svyato I.E. PhD[114], Sylaja P.N. MD, DM[115], Tao X. MSc[116], Thakur K.T. MD[117], Toni D. MD[118], Topcuoglu M.A. MD[119], Torales J. MD, MS[120], Towfighi A. MD[121], Truelsen T. MD, PhD[122], Tsiskaridze A. MD, PhD, DSc[123], Tulloch-Reid M. MD, DSc[124], Useche J.N. MD[125], Vanacker P. MD, PhD[126], Vassilopoulou S. MD, PhD[127], Vukorepa G. MD[128], Vuletic V. MD, PhD[129], Wahab K.W. MBBS, MPH, MSc, MD[130], Wang W. PhD[131], Wijeratne T. MD[132], Wolfe C. MD[133], Yifru M.Y. MD, MSc[134], Yock-Corrales A. MD, MSc[135], Yonemoto N. M[136], Yperzeele L. MD, PhD[126] , Zhang P. PhD[116]

Institutional Affiliations

1. Departamento de Enfermedades Neurovasculares, Instituto Nacional de Ciencias Neurológicas, Lima, Peru

2. Institute of Nutritional Sciences, University of Hohenheim, Stuttgart; Department of Radiation Oncology, Faculty of Medicine, Martin-Luther-University Halle-Wittenberg, Halle, Germany
3. School of Public Health, Addis Ababa University, Addis Ababa, Ethiopia
4. Department of Medicine, University College Hospital, Ibadan; University of Ibadan, Nigeria
5. National Center for Neurosurgery, Astana, Kazakhstan
6. Astana Medical University, Astana, Kazakhstan
7. Department of Neurology, Faculty of Medicine, Université de Parakou, Benin
8. Hospital de Santa Maria, University of Lisbon, Portugal
9. University of Ghana Medical School, Accra, Ghana
10. Department of Learning, Informatics, Management and Ethics (LIME), Karolinska Institutet, Stockholm, Sweden
11. Department of Neurology, Institute for Neurological Research, FLENI, Montañeses, Buenos Aires, Argentina
12. Medical University/University Hospital 'St. Marina', Varna, Bulgaria
13. Department of Linguistics & Communication Studies, Osun State University, Nigeria
14. Department of Molecular Medicine, College of Medicine and Medical Sciences, Princess Al-Jawhara Center for Genetics and Inherited Diseases, Arabian Gulf University, Manama, Kingdom of Bahrain
15. Escuela de Medicina San José Universidad de Costa Rica
16. Department of Medicine, Faculty of Medicine and Health Sciences, Universiti Putra, Malaysia
17. University of Nottingham, United Kingdom
18. Semmelweis University, Budapest, Hungary
19. San Gerardo Hospital ASST Monza, University of Milano Bicocca, Italy
20. Kaiser Permanente Bernard J. Tyson School of Medicine, California, USA
21. Universidad Peruana Cayetano Heredia, Lima, Peru
22. The Florey Institute of Neuroscience & Mental Health, University of Melbourne, Australia
23. Riga Stradiņš University and Riga East University Hospital, Riga, Latvia
24. Ministry of Health of Republic of Armenia, Yerevan State Medical University, Yerevan, Armenia
25. University Center of Primary Care and Public Health (Unisanté), Lausanne, Switzerland
26. Sveti Duh University Hospital, Department of Neurology, Zagreb, Croatia
27. Department of Medicine, School of Clinical Sciences at Monash Health, Monash University, Australia
28. Perugia University, Italy
29. Department of Pharmacology, Yong Loo Lin School of Medicine, National University of Singapore
30. Department of Neurology, New York University School of Medicine, New York, USA
31. Medical University of Gdansk, Poland
32. Hospital das Clínicas/University of São Paulo and Hospital Israelita Albert Einstein, São Paulo SP, Brazil
33. Institute of Public Health, University of Porto, Portugal
34. University of Palermo, Italy
35. Bunatian Institute of Biochemistry, Yerevan, Armenia

36. Royal Melbourne Hospital, Australia
37. International Institute for Brain Health, Zagreb, Croatia
38. Department of Neurological Surgery, School of Medicine and Public Health, University of Wisconsin, USA
39. Institute for Stroke and Dementia Research (ISD), LMU Hospital, Ludwig-Maximilians-University Munich; German Center for Neurodegenerative Diseases (DZNE, Munich); Munich Cluster for Systems Neurology (SyNergy), Munich, Germany
40. University of Melbourne, Australia
41. Division of Neurology Clinical Outcomes Research and Population Sciences (NeuroCORPS), Columbia University, New York, USA
42. Department of Neurology with Experimental Neurology, Charité-Universitätsmedizin, Berlin, Germany
43. Alneelain University, Khartoum, Sudan
44. Department of Neurology, University Hospital Bern, University of Bern, Switzerland
45. Neurosurgery Unit, CHUZ Abomey Calavi, Benin
46. Unidad de ACV, Instituto de Neurología, Hospital de Clínicas, Uruguay
47. I.M. Sechenov First Moscow State Medical University, Russia; WHO Regional Office for Europe, WHO European Office for the Prevention and Control of Noncommunicable Diseases, Moscow, Russia
48. Dijon Stroke Registry, University Hospital of Dijon, University of Bourgogne-Franche Comté, France
49. Research Center of Neurology, Moscow, Russia
50. Robarts Research Institute, Western University, Ontario, Canada
51. Department of Neurology, University of Amsterdam, The Netherlands
52. College of Medicine and Medical Sciences, Arabian Gulf University, Bahrain
53. Department of Physiotherapy, College of Medicine, University of Ibadan, Nigeria
54. Medical School, Faculty of Health and Medical Sciences, The University of Western Australia, Perth, Australia
55. Department of Medicine, Universiti Kebangsaan Malaysia Medical Center, Kuala Lumpur, Malaysia
56. Departments of Stroke Medicine and Cerebrovascular Internal Medicine, National Cerebral and Cardiovascular Center (NCVC), Osaka, Japan
57. Department of Rehabilitation Medicine, College of Medicine, Chungnam National University Hospital, Daejeon, South Korea
58. Department of Neurology, National Taiwan University Hospital, Taipei, Taiwan
59. SEARCH, Maharashtra, India
60. Al-Farabi Kazakh National University Kazakhstan
61. Mater University Hospital, Dublin, Ireland; University College Dublin, Ireland
62. Non-communicable Disease Department, World Health Organization, Geneva, Switzerland
63. Department of Neurology, Medical University of Innsbruck and VASCage, Research Centre on Vascular Ageing and Stroke, Innsbruck, Austria
64. Department of Neurology and Neurosurgery, University of Tartu, Estonia
65. National Institute for Stroke and Applied Neurosciences (NISAN), School of Clinical Sciences, Auckland University of Technology, New Zealand
66. Faculty of Medicine, University of Medicine, Tirana, Albania
67. Thungchang Hospital, Nan Province, Thailand

68. University of Glasgow, United Kingdom
69. Department of Neurology and Psychiatry, Clínica Alemana de Santiago, Facultad de Medicina Clínica Alemana Universidad del Desarrollo, Santiago, Chile
70. V.N. Karazin Kharkiv National University, Kharkiv, Ukraine
71. Department of Neurology, Linkou Chang Gung Memorial Hospital and College of Medicine, Chang Gung University, Taoyaun, Taiwan
72. Department of Medicine and Therapeutics, The Chinese University of Hong Kong
73. Department of Neurology, University of California, Los Angeles, USA
74. Heart and Stroke Foundation of Canada, Ottawa, Ontario, Canada
75. Masira Research Institute, Medical School, Universidad de Santander (UDES), Bucaramanga, Colombia
76. Department of Medicine, University of Sao Paulo, São Paulo, Brazil
77. epHealth Primary Care Solutions, Florianópolis, SC, Brazil
78. Department of Clinical Neurosciences, University of Cambridge, United Kingdom
79. Instituto Mexicano del Seguro Social (IMSS) HGZ 2, Aguascalientes, Mexico
80. Faculty of Medical Sciences, National Autonomous University of Honduras/WHO Collaborating Centre for Research and Community Intervention in Epilepsy
81. Department of Neurology, Janakpuri Super Speciality Hospital Society, Janakpuri, New Delhi, India
82. Department of Internal Medicine of Kyrgyz State Medical Academy, Bishkek, Kyrgyzstan
83. American Heart Association/American Stroke Association, Dallas, Texas, USA
84. Institute for Epidemiology and Medical Biometry, University of Ulm, Germany
85. Mater Misericordiae University Hospital, Dublin, Ireland; UCD School of Medicine, Dublin, Ireland; RCSI Medical School, Dublin, Ireland
86. Department of Community Medicine, School of Medical Sciences, Universiti Sains Malaysia
87. Department of Neurology and Psychiatry, Assiut University, Egypt
88. Emory University School of Medicine, Atlanta, Georgia, USA
89. Center for Stroke Research, Berlin; Department of Neurology, Charité-Universitätsmedzin, Berlin, Germany
90. Department of Medicine, Groote Schuur Hospital and University of Cape Town, South Africa
91. Primary Stroke Center, Pacífica Salud - Hospital Punta Pacifica, Ciudad de Panamá Servicio de Neurología, Complejo Hospitalario, Caja de Seguro Social, Panamá
92. Lagos State University College of Medicine/Lagos State University Teaching Hospital, Lagos State, Nigeria
93. Beni-Suef University Faculty of Medicine, Beni Suef, Egypt
94. Weill Institute for Neurosciences, University of California San Francisco, USA
95. Memorial Şişli Hospital, Istanbul, Turkey
96. Eskişehir Osmangazi University, Turkey
97. Turkish Neurological Society, Selcuk University Faculty of Medicine, Turkey
98. Head of Non-Communicable Diseases Department, Ministry of Health, Santiago, Chile
99. Health System Research Institute, Nonthaburi, Thailand
100. Institute for Neurorehabilitation and Evidence-based Practice ("An-Institut", University of Greifswald), BDH-Klinik Greifswald and Neurorehabilitation Research Group, Universitätsmedizin Greifswald, Greifswald, Germany
101. School of Medicine, University of Belgrade, Serbia

102. Department of Medicine, University of Otago, Wellington, New Zealand
103. PNS Shifa Hospital, Karachi, Pakistan
104. Department of Neurology, Donders Institute for Brain, Behaviour and Cognition, Radboud University Medical Centre, Nijmegen, The Netherlands
105. Miller School of Medicine, University of Miami, Florida, USA
106. Ballarat Health Service, Victoria, Australia
107. Department of Neurology and Neurosurgery, Faculty of Medicine, University of Chile, Santiago, Chile; Non-Communicable Diseases Department, Ministry of Health, Santiago, Chile
108. Stroke Outcomes and Decision Neuroscience Unit, Li Ka Shing Knowledge Institute, University of Toronto, Canada
109. Kwame Nkrumah University of Science and Technology, Kumasi, Ghana
110. Division of Neurology, Department of Medicine, McMaster University, Hamilton, Ontario, Canada
111. Department of Neurology, Yale School of Medicine & Yale New Haven Hospital, CT, USA
112. Neuro-intensive Care Unit, Institute of Neurosciences, Kolkata, India
113. Chulalongkorn University, Bangkok, Thailand
114. Moscow School of Management SKOLKOVO Russia
115. Sree Chitra Tirunal Institute for Medical Sciences and Technology (SCTIMST), Trivandrum, Kerala, India
116. The George Institute for Global Health, Beijing, China
117. Columbia University Irving Medical Center/New York Presbyterian Hospital, New York, USA
118. Department of Human Neurosciences, Sapienza University, Rome, Italy
119. Neurology Department, Hacettepe University Hospitals, Ankara, Turkey
120. Department of Psychiatry, School of Medical Sciences, National University of Asunción, Asunción, Paraguay
121. Los Angeles County Department of Health Services, California, USA
122. Department of Neurology, Copenhagen University Hospital, Rigshospitalet, Denmark
123. Department of Neurology, Ivane Javakhishvili Tbilisi State University, Tbilisi, Georgia; Head, Neurological Service, Pineo Medical Ecosystem, Tbilisi, Georgia
124. Caribbean Institute for Health Research, The University of the West Indies, Mona, Jamaica
125. Department of Radiology and Imaging Sciences, School of Medicine, Indiana University, USA; Hospital Universitario Fundacion Santa Fe de Bogota, Colombia
126. Antwerp University Hospital, Edegem, Belgium
127. First Department of Neurology, University of Athens Medical School, Eginition Hospital, Greece
128. University Hospital Dubrava, Zagreb, Croatia
129. Clinical Department of Neurology, Medical Faculty of UHC Rijeka, Croatia
130. Department of Medicine, University of Ilorin, Ilorin, Nigeria
131. Beijing Neurosurgical Institute, China
132. Department of Neurology, The Sunshine Hospital; The University of Melbourne, Victoria, Australia
133. School of Population Health & Environmental Sciences, Kings College London, United Kingdom

134. Department of Neurology, College of Health Science, Addis Ababa University, Addis Ababa, Ethiopia
135. Emergency Department. Hospital Nacional de Niños "Dr. Carlos Sáenz Herrera", CCSS, San José, Costa Rica
136. Department of Public Health, Juntendo University School of Medicine, Tokyo, Japan; National Center of Neurology and Psychiatry, Kodaira, Japan

SECTION 2. STROKE COST ESTIMATES

METHODOLOGY OF ECONOMIC ANALYSIS

The economic implications of a stroke can be broken down into (a) medical care and other costs that are incurred in the acute phase (e.g., hospitalisation expenses, ambulance fees, etc.); (b) costs incurred during the subsequent post-stroke care phase, that may involve rehabilitation, drug expenses, outpatient services, in some cases lasting several years; and (c) loss of income to the household of the person with stroke (and potentially production, if a working person cannot be replaced for a period of time) owing to the effect of strokes on morbidity and mortality. There may also be additional costs incurred by other parts of the economic system, including social services provided, and the excess burden (or efficiency losses) associated with the added taxes used to finance some of these consequences of stroke. The global economic impacts of stroke estimated in this supplementary section are focused on (a)-(c). In the case of (b), the cost estimates are the lifetime costs associated with the healthcare use and caregiving services to the person with stroke. In the case of (c), two main approaches are used to evaluate the economic loss. The human capital approach (see below) is used to assess the potential income loss to the household. However, this need not translate into an equivalent (social) production loss if the loss of work time due to a person being affected by stroke could be lowered by hiring a replacement. We used the "friction cost" approach (Koopmanschap et. al. 1995) to assess the potential production loss when replacement hiring occurs, as outlined in greater detail below.

We first estimate the lifetime direct medical care (including caregiving) costs associated with incident stroke cases.

Estimating Direct Medical Costs of Stroke

Step 1-1

Country-specific estimates of the number of incident and prevalent stroke cases, and numbers by type of stroke (ischaemic or haemorrhagic) were obtained from the GBD (Global Burden of Disease) database for 2017[1].

Step 1-2

The World Bank classification of countries by income was used to classify countries into (a) high income (HIC); (b) upper middle income (upper-MIC); (c) lower middle income (lower-MIC); and (d) low income (LIC).

Step 1-3 (Acute-phase and 1-Year Estimates of Stroke Costs)

We carried out an extensive search (PubMed and Google Scholar) of peer reviewed literature – but not a full systematic review – for studies on the costs of stroke. Search terms used included, stroke, economic burden, treatment costs, costs, expenditures, hospital costs, acute care, medical care, ischaemic stroke, haemorrhagic stroke, cost-effectiveness of stroke interventions, CVD, healthcare costs, etc., often in combination with individual country names. We limited our search to studies that used data from 2000 or later, and (ideally) published in the year 2005 or later. We were concerned that developments in stroke treatments over time would affect costs, and information from earlier years would not adequately reflect the direct costs of stroke at the present time. Our search led us to identify about 70 studies, of which 50 were ultimately read in full.

With two notable exceptions (Cameroon and India) most studies in low- and middle-income countries were focused on costs of hospitalisation and related services in the acute phase immediately following a stroke. In contrast, studies from high-income countries had a much larger share reporting 1-year costs following a stroke, including expenditures post-discharge. We could identify only 8 studies (all from high-income countries), that estimated lifetime healthcare costs (often including social care) following stroke, alongside 1-year costs.

Our review of the literature highlighted the very large cross-country (and sometimes within country) variations in costs during the acute phase and the 1-year period following the stroke. These variations were driven by multiple factors: patient composition (e.g., comorbidity status, or incident versus prevalent stroke cases), patients from a single (possibly high-end tertiary) hospital, inclusion (or exclusion) of social care following discharge, inclusion (exclusion) of informal care, incident versus prevalent stroke cases, stroke type, data from stroke registries and linked utilisation data versus self-reported healthcare use data from household surveys, and out of pocket spending (alternately insurer expenditures) as against all costs, whether subsidised or not. Importantly, very few of the stroke cost studies tried to address a key attribution problem: in a competing risks framework, attributing to stroke all the costs of health and social care utilisation following a stroke is likely to upwardly bias the costs of stroke. Comparability was also hindered by differences in the years for which information was available, and monetary denominations used (local currency, US$ or Euro, based on then prevailing exchange rates, or international dollars).

Next, we excluded studies relying on patients from a single hospital, or studies that focused on patients belonging to a sub-group with high healthcare needs (the exception being studies from LMICs, given the very studies from this group), or where a distinction between incident and recurrent cases was not possible. Where a breakdown by cost-type was provided, we excluded costs of informal care from our estimates, given the considerable variability in methodology used to estimate the costs of informal care (in our calculations, costs of informal were added on later, using methods described in the supplement to the manuscript).

Treatment cost estimates available in the literature were then adjusted in two ways: (a) First, cost estimates were brought forward to 2017 at current prices in the local currency. This involved adjusting for inflation (using the GDP deflator) and *for medical care inflation over and above the general inflation* (to take account of technological changes that increase the cost of medical services faster than other items). The latter was assumed to be 3% per year (or 0.03) irrespective of country; (b) using purchasing power parity for 2017 to convert the stroke cost estimates into 2017 international dollars, separately (where possible) for

ischaemic and haemorrhagic strokes. Tables 1 and 2 provide these estimates for high income countries and for low- and middle-income countries, for hospitalisation costs during the acute phase, and costs for the 1-year following stroke. These costs were separated for ischaemic and haemorrhagic strokes, where possible.

Table 1. Costs for Acute Stroke Care and Post-Stroke Care in the 12 months following a Stroke in Selected High-Income Countries

Country	Author	Price	Stroke type	Local currency		2017 I$	
				Acute	12months cost	Acute	12months cost
US	Girotra et al (2020)[2] (1)	2016 (USD)	Not specified	11,087	17,598	11,634	18,467
	Yousufuddin et al (2020)[3] (2)	2013 (USD)	Ischaemic stroke	18,154	-	22,681	-
			Haemorrhagic stroke	24,077	-	30,081	-
	Wang et al (2014)[4]	2008(USD)	Ischaemic stroke	18,963	-	28,289	-
			Haemorrhagic stroke	32,035	-	47,790	-
	Johnson et al (2016)[5]	2013 (USD)	Ischaemic stroke (Commercial)	19,682	24,737	24,590	30,905
			Ischaemic stroke (Medicare)	14,981	20,041	18,717	25,038
Canada	Goeree et al (2005)[6]	2004 (CAD)	Ischaemic stroke	19,026	46,775	26,849	66,009
			Haemorrhagic stroke	12,996	50,336	18,340	71,034
Australia	J Kim (2017)[7]	2013 (AUD)	Ischaemic stroke	13,925	-	11,080	-
			Haemorrhagic stroke	18,315	-	14,573	-
	Stroke Foundation (2020)[8]	2020 (AUD)	Not specified	-	30,442	-	19,180
Singapore	Ng et al (2015)[9]	2012 (SGD)	Ischaemic stroke	8,591	9,139	11,904	12,663
			Haemorrhagic stroke	18,706	19,900	25,919	27,574
	Chow et al (2010)[10]	2008 (SGD)	Not specified	6,783	-	11,180	-
South Korea	Cha (2018)[11]	2015 (USD)	Ischaemic stroke	2,630	4,766	3,788	6,315
			Haemorrhagic stroke	8,707	12,902	12,539	18,581
Belgium	Dewilde et al (2017)[12]	2014 (PPP)	Ischaemic stroke	16,954	33,147	20,100	39,299
Germany	Kolominsky-Rabas et al (2006)[13]	2004 (Euro)	Ischaemic stroke	6,731	18,517	15,747	42,227
Finland	Meretoja et al (2011)[14] (1)	2008 (Euro)	Ischaemic stroke	8,086	20,680	14,104	36,071
			Haemorrhagic stroke	9,875	28,620	17,224	49,920
Romania	Lorenzovici et al (2020)[15]	2017 (Euro)	Not specified	995	-	2,817	-
	Strilciuc et al (2021)[16] (2)	2019 (Euro)	Ischaemic stroke	-	5,227	-	13,559
Spain	Parody et al (2015)[17]	2004 (Euro)	Not specified	3,165 - 3,537	7,593 - 9,081	8,647 - 9,663	20,744 - 24,809
	Alvarez-Sabin et al (2017)[18] (2)	201 (Euro)	Ischaemic stroke	8,623	27,001	16,220	50,789
			Haemorrhagic stroke	7,126	28,916	13,404	54,392
Sweden	Persson et al (2012)[19] (1) (2)	2008 (Euro)	Not specified	10,500	19,000	16,689	30,200
	Lundstrom et al (2010)[20]	2003 (I$)	Ischaemic stroke	17,882	21,842	36,321	44,365
Netherlands	Van Eeden et al (2015)[21] (2)	2012 (Euro)	Ischaemic stroke	-	23,481		36,339
Switzerland	Mahler et al (2008)[22]	2003 (SFr)	Not specified	6,403	31,115	8,580	41,696
France	Chevreul et al (2013)[23]	2007 (Euro)	Not specified	6,950	16,686	13,283	31,890
Italy	Gerzeli et al (2005)[24] (2)	2001 (Euro)	Not specified	6,111	15,123	18,794	47,921
UK	Patel et al (2020)[25]	2014 (UK £)	Not specified	13,269	18,081	22,200	30,251

(1) Control, (2) Including informal care

Table 2. Costs for Acute Stroke Care and Post-Stroke Care in the 12 months following a Stroke in Upper Middle-Income Countries

Country	Author	Price	Stroke type	Local currency		2017 I$	
				Acute	12months cost	Acute	12months cost
China	Zhang et al (2019)[26]	CNY 2013	Ischaemic stroke	17,731	-	5,094	8,852
			Haemorrhagic stroke	38,758	-	11,134	19,349
	Yin et al (2018)[27]	CNY 2016	Ischaemic stroke	19,734	-	5,064	8,800
			Haemorrhagic stroke	37,719	-	9,679	16,821
	Huo et al (2017)[28]	CNY 2012	Ischaemic stroke	17,049	-	5,154	8,956
			Haemorrhagic stroke	34,937	-	10,561	18,354
	Yong et al (2016)[29]	CNY 2012	Ischaemic stroke	7,762 - 10,131	-	2,346 - 3,063	4,077 - 5,323
	Lu and Pan (2019)[30]	CNY 2016	Not specified	11,265	-	2,891	5,024
	Wei et al (2010)[31]	CNY 2007	Not specified	11,216	-	4,998	8,685
	Zhu et al (2020)[32]	CNY 2015	Ischaemic stroke	10,472	-	2,807	4,878
			Haemorrhagic stroke	26,847	-	7,196	12,505
Malaysia	Nordin et al (2012)[33]	MYR 2008	Ischaemic stroke	3,768	-	3,975	6,908
			Haemorrhagic stroke	3,774	-	3,982	6,919
Brazil	Vieira et al (2019)[34]	USD 2017	Ischaemic stroke	9,766	-	9,766	16,972
			Haemorrhagic stroke	21,790	-	21,790	37,868
	Safanelli et al (2019)[35]	USD 2017	Ischaemic stroke	5,020	-	5,020	8,724
			Haemorrhagic stroke	3,741	-	3,741	6,501
Argentina	Christensen et al (2009)[36]	PPP 2005	Ischaemic stroke	3,888	-	7,936	13,792
			Haemorrhagic stroke	12,285	-	25,077	43,580
Turkey	Asil et al (2011)[37]	USD 2005	Ischaemic stroke	1,467	-	3,190	5,544
			Haemorrhagic stroke	2,816	-	6,124	10,643
Vietnam	PI Tran (2015)[38]	PPP 2012	Ischaemic stroke	535	-	686	1,192
			Haemorrhagic stroke	637	-	816	1,419
Thailand	Sribundit et al (2017)[39]	THB 2008	Ischaemic stroke	42,400	-	5,165	8,975
Pakistan	Khealani et al (2003)[40]	Rs 2001	Not specified	70,714	-	11,731	20,386
Philippines	Diestro et al (2021)[41]	₱ 2017	Ischaemic stroke	29,648	-	1,529	2,658
			Haemorrhagic stroke	45,131	-	2,328	4,046
South Africa	Viljoen (2014)[42]	R 2012	Not specified	19,072*	-	4,575	7,951
Senegal	Kaur et al (2014)[43]	USD 2013	Not specified	416	-	947	1,646
Nigeria	Maredza and Chola (2016)[44]	USD 2013	Not specified	1,043 - 8,424	-	2,113 - 17,066	3,672 - 29,658
Cameroon	Aminde et al (2021)[45]	XAF 2017	Ischaemic stroke	-	932,700	-	4,006
			Haemorrhagic stroke	-	815,400	-	3,503
India	Kwatra et al (2013)[46]	INR 2011	Not specified	44,237	76,877	3,326	5,780

Step 1-4 (Representative Regional Costs for Stroke, and Attributable Stroke Costs)

Given the limited number of stroke studies, our strategy for constructing global estimates of the healthcare costs of stroke relied on constructing "representative" costs, based on country income-classification (see Table 3). Among high income countries (HICs), we limited our choice to studies that sought to address the risk of double counting (owing to individuals experiencing a stroke being potentially susceptible to other health conditions in a competing-risks framework) to achieve a better attribution of stroke costs. That left us with 4 studies that used a control group (one from US, one from Finland, and two from Sweden) to achieve a better estimate of costs attributable to stroke. The US study was missing social/institutional care costs (but it did provide an estimate of the likely bias), so its stroke cost estimates were appropriately scaled up. These 4 studies were used to construct the upper and lower bounds for stroke costs in the acute phase and in the 12 months following stroke in HICs.

Table 3. Cost per Incident Stroke Case for Global and Regional Estimates (in 2017 I$)

Region	***Acute Phase***	***Post-Stroke – 12 months***
HIC	Ischaemic stroke:11,634 – 14,104<br>Haemorrhagic stroke:11,634 – 17,224	Ischaemic stroke: 25,749 – 31,214<br>Haemorrhagic stroke: 25,749 – 38,119
UMIC	Ischaemic stroke:4,498 – 9,766<br>Haemorrhagic stroke: 3,861 – 21,790	Ischaemic stroke: 7,816 – 16,972<br>Haemorrhagic stroke: 6,710 – 37,868
LMIC & LIC	Ischaemic stroke:1,529 – 5,165<br>Haemorrhagic stroke: 2,328	Ischaemic stroke: 2,658 – 8,975<br>Haemorrhagic stroke: 4,046
China	Ischaemic stroke: 2,705 – 5,154<br>Haemorrhagic stroke: 7,196 – 11,134	Ischaemic stroke: 4,700 – 8,956<br>Haemorrhagic stroke: 12,505 – 19,349
India	1,996 – 3,468	3,326 – 5,780

Note: HIC = High Income Countries; UMIC = Upper Middle-Income Countries; LMIC = Lower Middle-Income Countries; LIC = Low Income Countries

In upper middle- and low-income countries, information was mostly missing for 1-year stroke costs. Data from a single Indian study (after excluding the costs of informal care and other adjustments) was used to scale up acute-phase hospitalisation costs to 1-year costs. With these modifications, data from China was used to construct a range of acute-phase hospitalisation and 1-year costs for that country; and data from India was used to construct a range for India. For upper-middle income countries, data from Argentina, Brazil, Malaysia, Turkey was used to derive a range, with costs at the lower end of the range comprising patients at public hospitals, and at the top end, private hospitals. For LMICs other than India, we excluded Vietnam and Pakistan that had abnormally low/high stroke costs. Data from other countries – Philippines, Cameroon, South Africa, and Nigeria – was used to construct the range of acute-phase and 1-year stroke costs.

The representative regional costs are reported in Table 3. Acute phase stroke treatment costs used for constructing direct cost estimates were based on the estimates reported in Table 3 for country income groups, China, and India. Given that the costs of stroke treatment tend to vary by type of stroke, haemorrhagic strokes were distinguished from ischaemic (and other) strokes. There are differences across countries in this proportion in the GBD database (the share of ischaemic strokes being higher in HICs and that of haemorrhagic strokes being higher in lower-MICs and LICs).

Although not depicted in Table 3, when estimating the global direct costs of stroke, we adjusted downwards the stroke cost estimates in HMICs, LMICs, China and India by 30%, to arrive at an estimate of attributable stroke costs (in a competing risks framework). This figure was roughly the proportional change achieved in the attributable costs of stroke, in the US study by Girotra et. al. (2020)[2] that used a control group when estimating stroke costs.

Step 1-5 Assumptions on Acute Phase Stroke Treatment Costs

It was assumed that all incident stroke cases need acute-phase treatment, reflecting the fact that the seriousness/acute nature of the condition is likely to result in the person affected (or their household) seeking treatment.

Step 1-6 Assumptions about Care Following the Acute Phase

It was assumed that stroke patients that died in any given year did not receive any post-acute phase care. Available literature suggests a 1-year mortality risk for stroke of 25% in HIC. We assumed a similar mortality rate for MICs and low-income countries (LICs) given the relatively younger ages at which stroke occurs in their populations. For the remainder (i.e., number of new stroke cases (Y) *MINUS* the number of new stroke cases who died (D), or (Y-D)), patients were assumed to use services consistent with the full 1-year costs in their country-income group.

Step 1-7: The Costs of Informal Care

Estimated costs of informal caregiving for stroke survivors are included as part of direct costs. A key challenge that needed to be addressed in this context was the lack of evidence on hours allocated to informal caregiving for stroke in low- and middle-income countries (LMICs), although it is well known to be significant (and typically provided by women) (Bettger et. al. 2019;[47] Brinda et. al. 2014;[48] Pandian et. al. 2016[49]). For high-income countries, relatively consistent information from studies in the US, France, Germany, and Australia was used to assume that roughly one-half of the stroke survivors needed caregiving, amounting to between 30-44 hours per week for each patient (Barral et. al. 2021;[50] Dewey et. al. 2002;[51] Joo et. al. 2014;[52] Skolarus et. al. 2016;[53] Australian Stroke Foundation 2020[8]), or approximately 20 hours per stroke survivor. Because the magnitude of the provision of informal care is dependent on country health- and rehabilitation systems, which are relatively weak in LMICs, we assumed that the informal caregiver hours for informal support in LMICs are 50% higher than their counterparts in high-income countries. In another (high) scenario, we also allowed informal caregiving hours in LMICs to be double that of their counterparts in high-income countries. Although evidence is obviously needed, we believe that even this is relatively conservative, given limited access to high quality stroke services in LMICs and the poor health outcomes that will likely result.

It is likely that individuals who need informal care following stroke may have ended up needing informal care even in the absence of the stroke, owing to underlying conditions that make them susceptible to other health shocks. Joo et. al. (2014)[52] estimated that weekly incremental informal caregiving hours attributable to stroke are 8.5 hours per patient, and that the economic value of informal caregiving per stroke survivor is $8,211 per year, of which $4,356 (53%) is attributable to stroke. For our global cost estimates, it was assumed that (a) informal care in high income countries was 10 hours per week per survivor; (b) informal care in countries other than HICs was 15 hours per week per survivor (low scenario); and (c) informal care in countries other than HICs was 20 hours per week per survivor (high scenario).

The valuation of time allocated to informal care has been done in a variety of forms in the literature, including in terms of opportunity cost of time (based on the work status of the individual providing caregiving), with unemployed individuals valued at zero, or at some low cost of leisure foregone; replacement cost (i.e., the hourly earnings of similar jobs in the economy), and so forth. The opportunity cost perspective that emphasises employment will undervalue women caregivers' contributions in LMICs given the low work participation rates of women often observed in LMICs. Moreover, work on family farms or other self-employment activities which are commonly prevalent do not provide readily calculated measures of income from work. Our preference was for a replacement cost approach. But with limited data available, we decided to use one-half of GDP per adult worker as the annual full-time cost of caregiving.

Step 1-8: Lifetime Direct Costs of Stroke per Incident Case

Most stroke cost studies, even in high-income countries, tend to emphasise 1-year costs. To measure lifetime costs of an incident single stroke case, we first obtained information on the relative magnitude of lifetime stroke costs to 1-year stroke costs among incident survivors for the countries for which data was available. Analysis of stroke costs based on longitudinal data from Australia, Belgium, Germany, Finland, France, Sweden, and the United Kingdom suggests that the ratio of lifetime costs of stroke per incident (first year) survivor ranges from roughly 2.1 to 4.0, and this was the range we used for our sensitivity analysis, with a base case as the mid-point of the range (Chevreul et. al. 2013;[23] Dewey et. al. 2003;[54] Dewilde et. al. 2017;[12] Gloede et. al. 2014;[55] Kolominsky-Rabas et. al. 2006;[13] Meretoja et. al. 2011;[56] Patel et. al. 2019;[25] Persson et. al. 2012[19]).

Second, we scaled up the 1-year cost estimates for countries by a factor of 2.1, 4.0 and the mid-point (3.05), under alternative scenarios. We acknowledge that these numbers could be even higher potentially in low- and middle-income countries with rapidly evolving health systems that allow for improved technologies and growing public insurance that could lower the patient out of pocket costs of health service use (but not overall resource use), although no data are available on this subject. This remains a limitation of the methodology adopted in this supplement.

Step 1-9: Estimates of the Global Cost of Treating Stroke.

This step essentially involved multiplying the incident stroke cases with estimates of (relevant) unit costs for acute stroke care and post-stroke care (and adjusting for lifetime stroke costs. This provided, for each income-region and globally, direct costs of stroke at the lower and the upper end. An estimate of the direct costs of stroke based on the mid-point of all the ranges of acute-phase and 1-year costs, informal caregiving time, and the ratio of lifetime to one-year costs of stroke was also constructed, and this provides the base case estimates reported in Table 4, in addition to the lower- and upper-bound estimates.

Table. 4: Direct Costs, Production Losses, and Household Income Losses due to Stroke in 2017 (Billions of 2017 I$ and Billions of 2017 US$) and as proportion of Global GDP

Region	Direct Costs						Production Loss (Friction Method)		Income Loss (Human Capital or Indirect Costs)	
	Base Scenario		Low Scenario		High Scenario					
	I$	US$	I$	US$	I$	US$	I$	US$	I$	US$
High-Income	213.45	210.50	127.79	127.87	316.48	308.50	29.43	25.39	335.33	289.10
Upper Middle-Income	144.54	91.30	62.16	37.05	260.72	168.98	25.98	12.77	490.21	241.00
Lower Middle Income	31.50	11.50	14.68	4.72	54.66	21.19	2.73	0.82	144.53	43.60
Low Income	3.52	1.49	1.57	0.55	6.24	2.85	0.10	0.04	5.49	2.00
Global Total	393.01	314.79	206.20	170.19	638.10	501.52	58.14	39.02	975.56	575.70
As % of Global GDP	(0.31%)	(0.39%)	(0.16%)	(0.21%)	(0.51%)	(0.63%)	(0.05%)	(0.05%)	(0.77%)	(0.73%)

Note: China is categorised as an Upper Middle-Income country; and India under Low and Middle-Income Countries. Numbers in parentheses indicate share of GDP in percentages.

Next, we turn to the methodology for the estimates of income (production) losses.

Estimation of Income Losses

Two approaches were used. First, income losses were estimated for incident stroke patients during the reference year (2017). Note that this is not the same as production losses to the economy or employers, which is better captured by other approaches, such as the friction cost method described below. However, income losses are likely to be more effective at capturing economic well-being of affected households and implications for equity relative to methods based on production losses.

Income losses for stroke patients were estimated by: (a) evaluating the number of years of working life lost due to stroke; and (b) the earnings losses per year during the working life lost. A discount rate for future income losses of 5% was assumed (this is typically around the mid-point of the discount rates used in the economic literature on evaluation, which range from 3%-10%). Notice that current real rates of interest (often used to guide choice of discount rates) are close to zero globally. Using a discount rate of 5% would make the estimates of income losses reported here, conservative. The annual rate of growth of real wage in the future was taken to be the same as the annual rate of growth of real GDP per working age person (15-64) in the period 2010 to 2017. Log-linear regression models were run to estimate these rates separately for each country, using annual data from the World Development Indicators database of the World Bank.

Estimation of number of working years lost for people with stroke

It was assumed that people retire at 65 years in all countries. Data on the incidence and prevalence of strokes for the age-group 15-64 years was obtained from the GBD database.

These numbers, available in 5-yearly intervals (15-19, 20-24, …etc.), were used to first estimate the average age of an individual below 65 who had a stroke in 2017.

Step 2-1: Estimation of average age at which stroke occurred among individuals less than 65 years in 2017

If the number of strokes in 5-yearly groups in the age-range 15-64 is denoted by $\pi_1, \pi_2, \pi_3, \dots \pi_{10}$, then the average age of individuals aged 15-64 experiencing a stroke in 2017 is Mean Age, where

$$Mean\ Age = \frac{17\pi_1 + 22\pi_2 + 27\pi_3 + \cdots + 62\pi_{10}}{\pi_1 + \pi_2 + \pi_3 + \cdots + \pi_{10}}$$

The coefficients 17, 22, … etc. denote the mid-point of each 5-yearly age interval in 15-64. Here, the denominator is the number of incident stroke cases in ages 15-64 for each country and can be denoted as N_{15-64}, where:

$$N_{15-64} = \pi_1 + \pi_2 + \pi_3 + \cdots + \pi_{10}$$

This calculation assumes that stroke patients are uniformly distributed within each age interval and was used to estimate Mean Age at stroke for the age group 15-64 years for each country in the GBD dataset.

Step 2-2: Life expectancy of an individual aged 15-64 following stroke

Given N_{15-64} as the number of new stroke cases in the age group 15-64 in 2017, let P_{15-64} be stroke prevalence in the 15-64 age group. In steady state, an approximation to the expected number of years lived (E) following a stroke in this age group can be calculated for each country as

$$E = \frac{P_{15-64}}{N_{15-64}}$$

This formula yields a lower bound to the expected years of survival because some individuals will drop out of this age group over time by moving to 65+, and not because they died. This downwardly biases the estimate of the expected years of life following a stroke (and hence the income loss upwards) but the bias to income losses will be small due to discounting, as the move to the 65+ age category will occur several years down the road.

Step 2-3: *Number of working years lost*

For purposes of estimation of working years lost, it was assumed that all strokes in the age group 15-64 occurred at the mid-point of 2017 in each country and at the mean age E for the age group 15-64 in that country.

It was assumed that an individual survivor with stroke would roughly work about 50% of full-time. If the expected number of years of survival E were such that $Mean\ Age + E \geq 65$, then the per person loss in working years (L) in the 15-64 age group would be

$$L = 0.25 + (0.5)(65 - Mean\ Age - 1)$$

The loss in working years would be different if the expected number of years lived following a stroke (E) were such that the person died before 65. Here, following death, the entire working year would be lost for all subsequent years up to age 65.

Thus, if $Mean\ Age + E < 65$

$$L = 0.25 + (0.5)E + (1.0)(65 - Mean\ Age - E - 1)$$

Step 2-4: Estimating income losses for people with stroke in each country

To illustrate the method used, assume that the wage per person in 2017 is W and the growth rate of wage (in 2017 prices) is β. Assume that the discount rate is R.

Example 1:

Suppose for a given country X, the mean age at stroke for people under 65 is given by $Age_X = 45;\ and\ that\ E_X = 16$.

Then, the income loss per person is 1/4 of the wage in the first year, half the wage in the following 15 years, and the full wage in the remaining 4 years (till age 65 is reached). The discounted income loss is:

$$Income\ Loss = \frac{W}{4}\frac{(1+\beta)}{(1+R)} + \frac{W}{2}\frac{(1+\beta)^2}{(1+R)^2} + \cdots + \frac{W}{2}\frac{(1+\beta)^{16}}{(1+R)^{16}} + W\frac{(1+\beta)^{17}}{(1+R)^{17}} + \cdots + W\frac{(1+\beta)^{20}}{(1+R)^{20}}$$

Example 2:

Consider country Y, where $Age_Y = 55;\ E_Y = 16$

Then income loss per person is given by

$$Income\ Loss = \frac{W}{4}\frac{(1+\beta)}{(1+R)} + \frac{W}{2}\frac{(1+\beta)^2}{(1+R)^2} + \cdots + \frac{W}{2}\frac{(1+\beta)^{10}}{(1+R)^{10}}$$

Here only 10 years of work-life are lost. For the years for which the person lives beyond 65, there is no income loss.

It is again worth underlining that income losses cannot be taken to imply productivity losses or national income losses in any strict sense of the term, but there are equity implications, since some lose out and others gain. Previous theoretical work has also argued that household economic losses can be interpreted as a lower bound for the value of a statistical life.

Step 2-5 Method used to calculate (expected pre-stroke wage) W_i and (expected post-stroke wage) W_s^i

Even if a person is of working age, they may not have a job. To account for this, the estimates used here relied on the notion of 'expected wage', and the assumption that the likelihood of a person in the age-group 15-64 holding a job was equal across ages and gender and given by the ratio of workers (or employed) in the 15-64 age group to the working age (15-64) population. This number was obtained from the World Development Indicators database (World Bank 2021).

Denote the likelihood of any person aged 15-64 years holding a job in country $'i'$as δ_i, and that every person in that group has an equal likelihood of being employed. Then a reasonable proxy for the *expected* wage for an individual is W_i, which depends on the share of people who work and the output per worker.

$$W_i = \delta_i \text{ x} \frac{GDP_i \text{ } in \text{ } 2017 \text{ } International \text{ } Dollars}{Total \text{ } Number \text{ } of \text{ } Workers \text{ } in \text{ } 'i'} = \frac{GDP_i \text{ } in \text{ } 2017 \text{ } International \text{ } Dollars}{Total \text{ } Population \text{ } Aged \text{ } 15-64 \text{ } in \text{ } 'i'}$$

Data on GDP in international dollars (current) was obtained from the World Development Indicators database.

For W_s^i it was assumed that the functional status of individuals after their stroke was less than before their stroke, noting that there are both minor and major strokes, and not all cases will completely exit from the workforce. Previous work, mainly from HICs and a small number of middle-income countries, suggests that between 40%-75% of working age stroke survivors return to employment over time.[57-61] As well as lower workforce participation rates compared to counterparts who did not experience a stroke, the wages of stroke survivors who return to work are also lower.[62] Based on this evidence we assume that employed individuals who first had a stroke in 2017 see a halving of their income for the rest of their working life (those who die lose their full pre-stroke income). So, the working assumption was $W_s^i = 0.5W_i$.

Step 2-6 Global Income Losses

These are estimated by multiplying for each country the number of incident cases of stroke by the income loss per incident stroke case, Y_i among people in the age group 15-64 years. Alternative ways to estimate income losses exist. For example, data on the national (regional) age distribution of incident stroke cases, combined with expected years of survival for each age group (in that distribution) could also be used to construct a measure of national (global) economic loss. The approach used in this note, by appealing to steady state considerations, enables a more direct method to estimate income losses.

Estimation of Production Losses: Friction Cost Approach

The basic idea under the friction cost approach is that the method used to assess the income loss to an individual (human capital approach) does not work as well to assess production losses to society. This is because in a setting with unemployment, persons leaving work permanently (via death or disability) or temporarily (sick leave) can be replaced by others who may be available. From this perspective, what matters for costs is the "friction period", the time between the absence from work of the sick person (in this case the individual with stroke), and any costs related to the hiring or training of the replacement. Beyond that no productivity loss is envisaged. One exception is when an already employed person replaces the one who is sick, thereby creating another vacancy elsewhere, and so on. This suggests that a more complete friction cost approach ought to the account for the cumulative sum of friction periods resulting from a vacancy generated by death, disability, or temporary absence from work.

The major challenge in applying the friction cost approach is lack of data, especially in LMICs, related to (a) the length of the friction period – the time over which the person with stroke (mortality or disability) is replaced by another, and thus the period over which production is lost; (b) the cumulative nature of friction periods, as when people move from an employed

status to the vacancy created by the person affected by stroke, thus creating a vacancy in their existing position; and (c) accounting and valuing for loss of leisure time (when an individual moves from an unemployed state to fill the vacancy generated by the person with stroke). For instance, a recent study for Malaysia estimated the costs of CVD using a friction cost approach but relied almost completely on data from European countries for (a) and (b) (Ministry of Health Malaysia 2020).

Step 3-1: Estimating the Friction Period

As noted above data on friction periods is rarely available in LMICs. Studies using European data work with a friction period of 60 days, sometimes higher. In the United States, available estimates suggest that costs of replacing workers amount to roughly 21% of annual employee salary, or equivalent to roughly 55 days (Boushey and Glynn 2012).[63] In India, a study of the Information Technology (IT) industry estimated costs of replacing workers to be approximately 10% of annual employee salary, or effectively about 26 days of work (Gochhayat and Nanda 2011),[64] reflecting the highly competitive nature of this industry and its attraction to employees. Presumably, one can assume that similar friction periods apply to small enterprises in the informal sector that hire employees on a wage basis, and in elementary occupations, such as unskilled agricultural work, where substitutes are readily available.

It is not obvious, though, that estimated costs of worker replacement in the literature, which are typically calculated for formal sector jobs, can be readily translated to all categories of informal sector work, especially self-employment, including sole proprietorship. For instance, sole proprietorship has to do with entrepreneurial skills, and these are not necessarily replaceable, and the human capital approach is likely to be a better indicator of the production loss here. Nor is it obvious, given the onerous labour laws and hiring practices in many LMICs – that all formal sector jobs operate on the same principles as the software industry – and indeed the friction periods and hiring costs will likely be much higher for government jobs, a major source of employment. But paucity of data makes accounting for these possibilities in our calculations difficult.

Here we undertake a particularly simple version of friction cost analysis. In the absence of data on cumulative friction periods arising from a vacancy, we used 60 days as the applicable friction period for high income countries, and 30 days for low-income countries (based on the Indian evidence). Because we did not include the added costs of leisure foregone by the new workers or allow for a sequence of friction periods arising from the filling of vacancies arising from stroke, our estimates are likely to constitute a lower bound to production losses from a more complete friction costs approach. This is a limitation of our analysis.

Step 3-2: The Annual Output per Worker

As in the case of the human capital approach, we assumed the annual output per worker to be GDP per member of working age group, that is W_i for a given country i . The resulting estimates of productivity losses are provided in Table 4, and provide a lower bound for production losses, if estimates based on the human capital approach are taken as an indicator of production losses as well.

SUMMARY

Table 4 summarises the main findings for global treatment expenditures incurred in the first 12 months of stroke. These point to health expenditures on incident stroke cases of about I$ 393 billion, or about 0.3%, of global GDP measured in I$, in 2017. Given that the global economy was growing annually at about 3.5% for much of the decade, healthcare costs for stroke would have accounted for almost one-tenth of annual growth in that year. Table 4 also provides a low-end and high-end range of direct cost estimates for stroke, from 0.16% of global GDP to 0.5% of global GDP.

Table 4 also provides estimates of output losses using two different approaches. The lower bound estimates rely on the friction cost method which is well suited to capturing production losses. Compared to direct costs, these estimates point to limited effects on global output, approximately 0.05% of global GDP. The upper-bound estimate relies on the human capital approach and suggests output losses of almost I$976 billion in 2017 (or about 0.8% of global GDP). In settings with high levels of unemployment and underemployment, the human capital approach is likely to yield overestimates of production losses. However, it is still a useful measure of income losses to individuals affected by stroke and their households, with important welfare and equity implications.

Our economic analysis was designed to develop an economic case for pragmatic solutions. We estimated the global financial costs of providing care to patients with stroke immediately following admission, in the period 12 months following, lifetime costs, and associated income losses. We obtained data from the GBD study on the number of new stroke cases, and deaths from stroke in 2017,[1] and combined these figures with available estimates of the costs of treatment. Our estimates suggest that stroke-related treatment and rehabilitation costs ranged from approximately I$206 billion to I$638 billion among new stroke cases in 2017, or between 0.16% and 0.50% of global GDP measured in international dollars. The low- and high-ends of the range of aggregate stroke costs primarily reflect the wide range of stroke treatment costs across countries, and the considerable variation in the components included in the costs of post-stroke care reported in the literature. Roughly one-half of the stroke treatment expenditure occurred in HICs, reflecting both the higher incidence of strokes as well the considerably larger costs of treatment and rehabilitation in these regions. Another 35%-40% of the spending was in upper middle-income countries, and the rest, about 5%-10% of global treatment costs and post-stroke care spending, was in LMICs.

In addition to treatment costs, there was also a significant cost in terms of income losses due to premature death and disability among stroke affected individuals (and their households), although the adverse effects on national output are less apparent. Previous work, mainly from HICs and a small number of middle-income countries, suggests that between 40%-75% of working age stroke survivors return to employment over time.[57-61] As well as lower workforce participation rates compared to counterparts who did not experience a stroke, the wages of stroke survivors who return to work are also lower.[62] We estimate that the discounted lifetime economic losses to households with incident stroke cases in 2017 amount to I$976 billion globally (or 0.8% of global GDP). Roughly one-third of the global economic losses from stroke occurred in HICs and another 50% in upper middle-income countries. Income losses from stroke in LMICs and LICs accounted for only 15% of the global total, primarily because of their much lower income levels. The share of income losses accounted for between three-fifths to four-fifths of the total global economic costs of stroke.

With significant treatment, rehabilitation, and other direct costs that amount to about 10% of the economic gains worldwide, interventions that can help prevent or reduce stroke incidence and/or mortality by small numbers can potentially yield very large economic returns.

SECTION 3. PRIMARY STROKE PREVENTION and SDGs

The United Nations (UN) targeted Sustainable Development Goal (SDG) 3.4 estimated that a one-third reduction of premature mortality from stroke through better acute care and prevention would avert 2.4 million deaths in 2030.[65] This estimate is based on the current trend in the increase of the number of new strokes (2.4% per year), prevalent strokes (2.9% per year), deaths from stroke (1.5% per year) and disability due to stroke (1.0% per year, as measured by disability adjusted life years - DALYs).[66] It is projected that without intervention, in 2030 there will be 14.2 million people with new strokes, 123.0 million people living with stroke aftermath, 7.2 million stroke- related deaths, and 153.2 million DALYs due to stroke.. However, according to the most recent GBD Study estimates,[67] in 2019 we already had 12.2 million people with new strokes, 101.5 million stroke survivors, 6.6 million stroke related deaths and 143.2 million DALYs due to stroke. Our proffered solutions are not only targeted at mitigating this projected burden of stroke, but at preventing economic losses arising therefrom.

In a broader context, investing in prevention of CVDs is integral to achieving UN defined SDG target 3.4 and to progress towards at least nine SDGs.[68] Many countries have implemented cost-effective interventions at low levels, so the potential to achieve these targets and strengthen national income by scaling up these interventions is enormous.[68] A strengthened effort across multiple sectors with effective economic tools, such as price policies, subsidised access to preventative services and insurance, is necessary. Stroke is heavily clustered in people with low socioeconomic status and is an important cause of medical impoverishment,[68] thereby exacerbating economic inequities within societies. Thus, stroke is a barrier to achieving SDG 1 (No Poverty), SDG 2 (Zero Hunger), SDG 4 (Education), SDG 5 (Gender Equality), and SDG 10 (Reduce Inequalities). Productivity gains from preventing and managing stroke will contribute to SDG 8 (Economic Growth). SDG 11 (Sustainable Cities and Communities) and SDG 12 (Responsible Consumption and Production) offer clear opportunities to reduce the burden of stroke and other NCDs and to create sustainable and healthy cities.[68]

SECTION 4. TANGIBLE SOLUTIONS READY FOR GLOBAL IMPLEMENTATION

The 2019 American ACC/AHA Guideline[69] on the primary prevention of CVD emphasises the importance of promoting a healthy lifestyle throughout life, evaluation of social determinants of health to inform treatment decisions, screening of adults aged 40 to 75 for 10-year CVD risk to inform pharmacological therapy, healthy diet (low sodium, low fat, low cholesterol and low glycaemic index), adequate physical activity (150 min per week of moderate-intensity or 75 min per week vigorous-intensity physical activity), smoking cessation as well as adequate control of low-density lipoprotein (LDL) cholesterol and blood pressure. They also acknowledge the importance of a combination of population-wide preventative strategies

with a comprehensive person-centred approach that addresses all of an individual's lifestyle habits and estimated risk of a future CVD event.

As stroke shares many risk factors with other major NCDs (such as ischaemic heart disease, diabetes mellitus, vascular dementia, and some types of cancer), it is logical that to be most cost-effective, primary stroke prevention strategies must be integrated into the overall strategies for prevention of all of these NCDs to achieve the suggested global targets in reducing NCD mortality and prevalence of risk factors. Only by joining forces with other interventions for NCDs prevention will stroke prevention have its full impact.[70-72] The Global Alliance for Chronic Diseases[73] is a good example of such an integrative approach. In our paper we tried to provide evidence-based pragmatic solutions on strategies for primary stroke prevention, including a critical review of existing primary prevention strategies and current guidelines, economic analysis, and identification of gaps in primary stroke prevention (Figure 2).

Figure 2. Four steps to derive tangible solution for primary stroke prevention recommendations

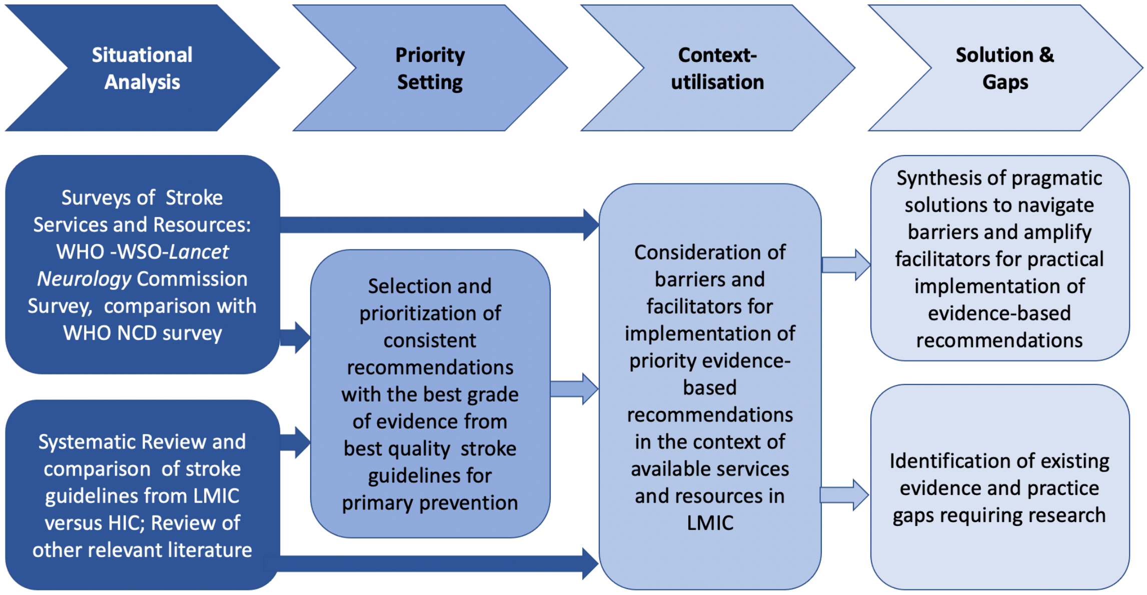

On the individual level in the general population, the suggested target SBP goals are: <120 mm Hg for people younger than 50 years, <130 mm Hg for people aged 50-74 years, and <140 mm Hg for people aged 75 years and older.[74] It was estimated that healthy lifestyle modifications on the individual level can reduce stroke incidence by about 40-50%,[75,76] but the effect of such modifications on the population level (shifting the lifestyle risk profile of the community towards healthier lifestyle) could be substantially greater.[77] A similarly large effect of lifestyle modification (healthy diet, adequate physical activity, smoking cessation etc.) was suggested for reducing the risk of recurrent stroke.[78] As many lifestyle habits are set early in life, culturally appropriate education about healthy lifestyles should be incorporated into standard education curricula and started early in life, with reinforcement across the lifespan.[79] These preventative strategies should be facilitated by adequate stroke education campaigns that take into account not only cultural and subcultural differences and beliefs of people of various races and ethnicities[80] but also significant geographical differences in the

lifetime risk of stroke[79] and its risk factors.[81] There is also evidence that the intensity of primary stroke prevention should not be reduced in older people.[79]

Although effective primary stroke prevention must include a combination of population-wide and individual-based preventative strategies,[82] the World Stroke Organization and World Heart Federation emphasise that priority (including resource allocation) should be given to the population-wide strategies (figure 3).[83,84] Given the fact that the majority of stroke burden (60-70%) across all countries in the world is associated with elevated systolic blood pressure (SBP) and unhealthy lifestyle risk factors, such as smoking, obesity, low physical activity and poor diet (including excessive salt, sugar and alcohol intake;),[75,81,85-90] reducing exposure to these risk factors and treating hypertension should be the priority targets for both population-wide and individual-based preventative interventions for primary (and also secondary) stroke prevention.[81] Additional priorities for setting up population-specific stroke preventative strategies should be informed by the most recent estimates of the population-attributable risks, which are currently available for 204 countries.[67]

Figure 3. WSO Declaration on primary stroke and dementia prevention (modified from Brainin et. al. The Lancet Neurology 2020,[84] with permission)

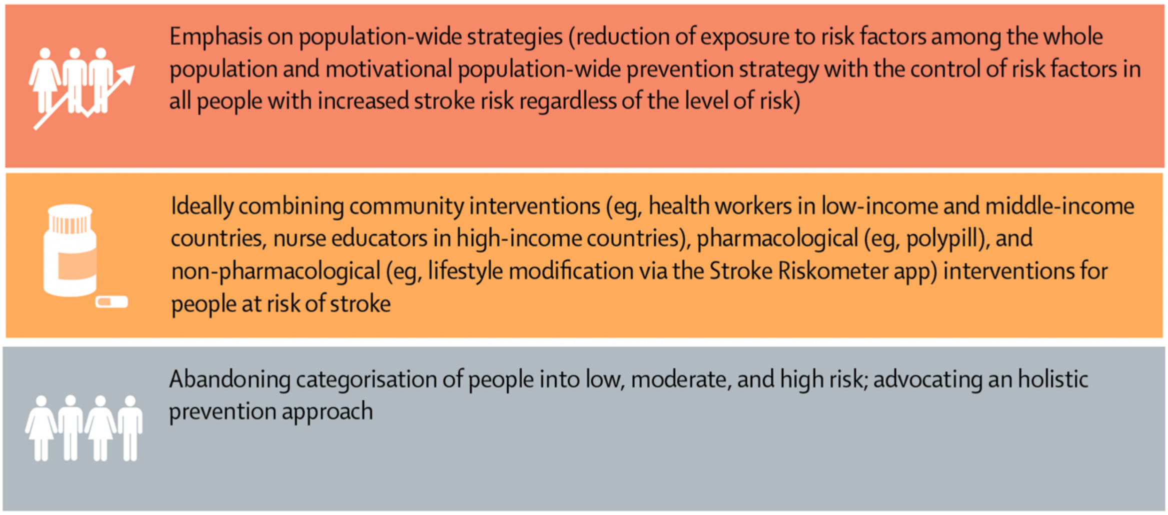

eHealth technologies

Mobile technology applications, also known as apps, provide a novel way to address current gaps in primary prevention. There was a massive rise in recent years in mobile data usage and it was expected to grow by more than 50% by 2020.[91] Mobile health (mHealth) has become an increasingly popular mode of healthcare delivery by providing a novel way to address current gaps in primary prevention. Advances in mobile devices coupled with high-speed internet means phones are becoming more than communications devices and are acting more as hubs for educational purposes, as well as portals that other devices can communicate through. In 2017 there were approximately 325,000 mHealth apps, with an annual download of >3.7 billion. There is widespread evidence of the feasibility and efficacy of apps for behaviour change across a range of health behaviours [92,93] and outcomes including clinically meaningful reductions in blood pressure.[94] There also are a lot of apps aimed at generic health factors such as diet and exercise that decrease users' CVD risk, despite not being explicitly marketed for this purpose. Activity trackers like Fitbit™ have huge user bases (recent filings indicate there are approximately 19 million registered users with over 9 million "active" users [95]), and research indicates that usage of wearable devices such as these does

show an increase in physical activity.[96,97] Similarly, diet trackers and calorie counters such as MyFitnessPal™ also show efficacy when used on a regular basis.[98] Similar generic apps are also available for blood pressure,[99] smoking cessation[100,101] and other associated risk factors. However, there is a lack of proven effective, valid, internationally endorsed, comprehensive (providing not only risk estimates and their monitoring but also a user's risk profile and specific evidence-based recommendations for controlling those risks) and free of charge apps for primary prevention of specific major non-communicable disorders. To the best of our knowledge, in primary stroke prevention there is only one app that meets most of these requirements: the Stroke Riskometer™ app.

The Stroke Riskometer™ app is a free app owned and copyrighted by Auckland University of Technology, New Zealand.[102] The Stroke Riskometer™ algorithm was derived from the Framingham Stroke Risk Score[103] prediction algorithm and enhanced to include several additional major risk factors shown to be important for stroke, largely based on the INTERSTROKE study.[86] The Stroke Riskometer has been found to be comparable in performance for stroke prediction with FSRS and QStroke.[104]

The app not only calculates 5- and 10-year absolute and relative risk of stroke occurrence but also provides patient-tailored recommendations for primary stroke prevention, which are based on the internationally recognised primary stroke and CVD prevention guidelines.[69,105] It incorporates several evidence-based tools to promote behaviour change aligned with internationally recognised stroke prevention guidelines.[69,105] These include:

1) Provision of feedback on *absolute* risk of stroke within the next 5 to 10 years *and* compares a person's *relative* risk with those of a person of the same age and sex without risk factors. This approach has been demonstrated to motivate behaviour change when used in conjunction with other methods.[106,107]
2) Employs *tailored self-management* strategies including goal setting to engage the person in behaviour modification.[108]
3) Includes *information on stroke risk factors and warning signs* aligned with the internationally relevant Face, Arm, Speech, Time (FAST) international mass media campaign.
4) Uses *reminders*, known as "push notifications", to prompt users to achieve their goals. Such reminders have been shown to increase adherence to programmes.[109]

This mobile app applies "motivational mass strategy for primary stroke prevention"[110] regardless of the level of stroke/CVD risk,[72,83] which is complementary to the high CVD risk strategy recommended by the WHO.[111] It has been endorsed by the World Stroke Organization, World Federation of Neurology, World Heart Federation, European Stroke Organisation and a number of national stroke organisations, including Australian Stroke Foundation, French Neuro-Vascular Society, and Chinese Stroke Society to name a few. The Stroke Riskometer™ app, its advantages and disadvantages and use/place in primary stroke/CVD prevention were discussed at various conferences and intensively reviewed,[72,80,84,102,104,106,107,110,112-129] and the app is recommended for the world-wide use.[130-132] At the recent Latin American summit of Health Ministers, the Stroke Riskometer™ app was recommended for implementation across all Latin American countries.[114]

Being available in 19 languages (English, Bengali, Bulgarian, Chinese [Mandarin], Croatian, Czech, French, German, Greek, Hindi, Italian, Malay, Nepali, Portuguese, Brazilian-Portuguese, Russian, Spanish, Swedish, Thai) this free mobile stroke education and

prevention tool is already available to 5.3 billion people in their native languages. It has already been downloaded by >200,000 people from 78 countries.

A desktop version of the Stroke Riskometer™ app – called the PreventS© webapp - takes only a couple of minutes to complete by a clinician. Like the Stroke Riskometer app it also provides patient-tailored recommendations and risk monitoring for primary stroke prevention and other major NCDs (e.g., ischaemic heart disease, type II diabetes mellitus, renal disease, vascular dementia and some types of cancer) for the individual patient, but unlike the Stroke Riskometer app, these recommendations can be further edited by the clinician to make it more specific and appropriate for the individual. The clinician also has the option of printing out the patient's summary (including recommendations and risks dynamic), saving the data for further monitoring of the risks progress, or sharing it with another clinician. As such, the PreventS© webapp is the only decision-making tool for integrative primary stroke and other major NCDs currently available for clinicians. Further details on the Stroke Riskometer™ app and PreventS-MD™ webapp can be found on the Auckland University of Technology website.

SECTION 5. GLOBAL STROKE CONTROL OBSERVATORY AND BURDEN REDUCTION ECOSYSTEM (G-SCORE)

Figure 4. Global Stroke Control Observatory and burden Reduction Ecosystem (g-SCORE)

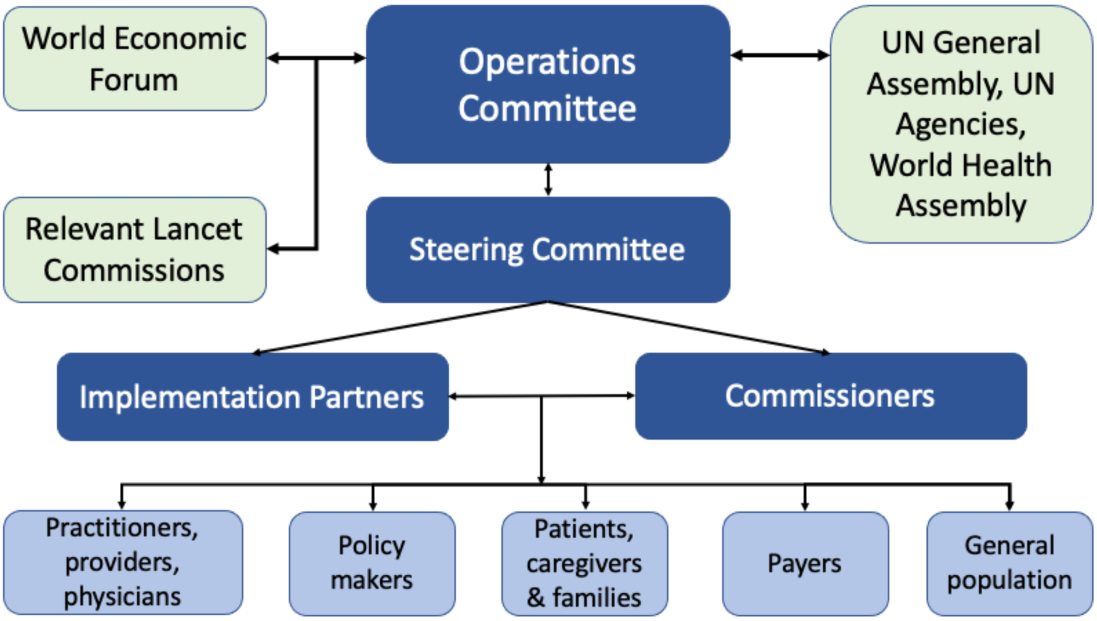

SECTION 6. FIGURES

Supplement Figure 5. Infographic: the global impact of stroke and stroke risk factors[67,79] (estimates of the stroke cost were derived from the current publication)

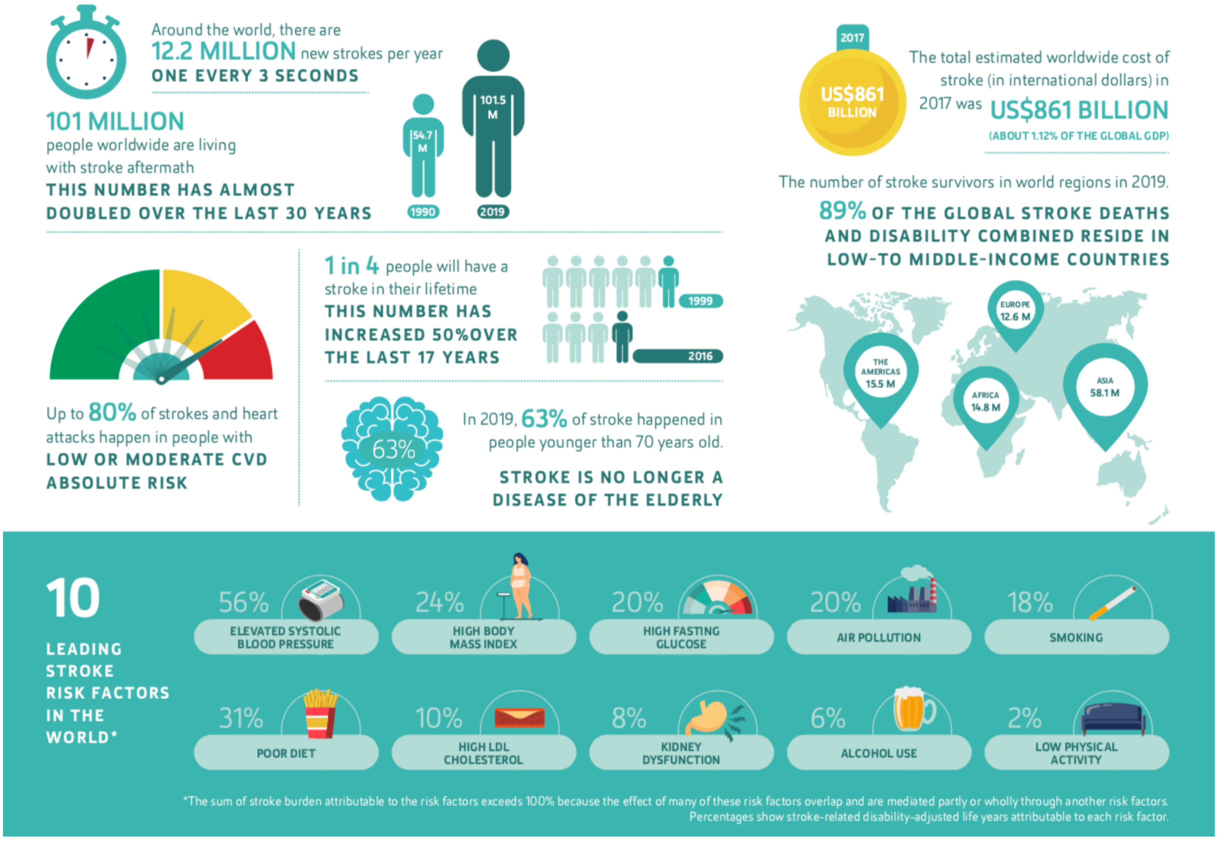

Supplement Figure 6. Outline of the PreventS-MD™ cloud-based platform for clinicians. The PreventS-MD™ algorithms for calculation absolute and relative risks of stroke are based on the validated and internationally endorsed Stroke Riskometer™ app.[107,110,133]

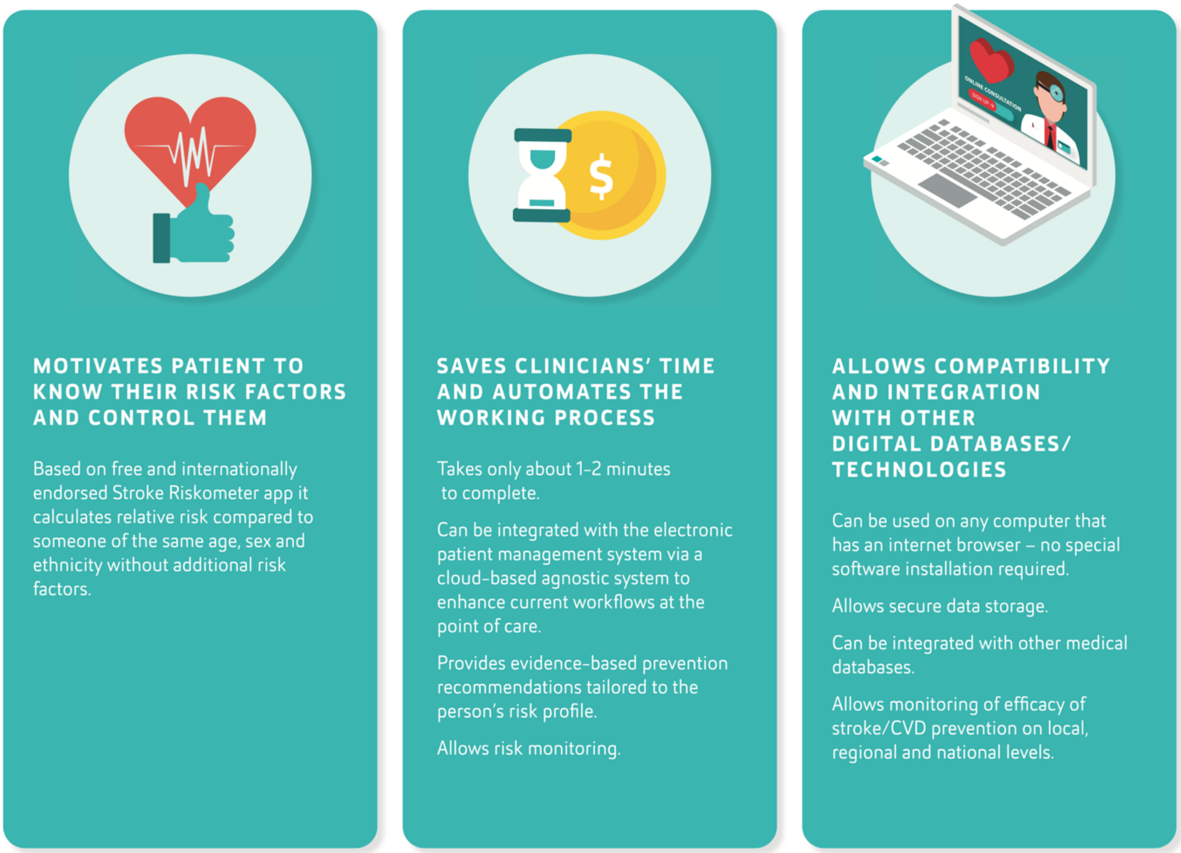

REFERENCES

1. Krishnamurthi RV, Ikeda T, Feigin VL. Global, Regional and Country-Specific Burden of Ischaemic Stroke, Intracerebral Haemorrhage and Subarachnoid Haemorrhage: A Systematic Analysis of the Global Burden of Disease Study 2017. *Neuroepidemiology* 2020; **54**(2): 171-9.
2. Girotra T, Lekoubou A, Bishu KG, Ovbiagele B. A contemporary and comprehensive analysis of the costs of stroke in the United States. *J Neurol Sci* 2020; **410**: 116643.
3. Yousufuddin M, Moriarty JP, Lackore KA, et al. Initial and subsequent 3-year cost after hospitalization for first acute ischemic stroke and intracerebral hemorrhage. *Journal of the Neurological Sciences* 2020; **419**.
4. Wang G, Zhang Z, Ayala C, Dunet DO, Fang J, George MG. Costs of hospitalization for stroke patients aged 18-64 years in the United States. *Journal of stroke and cerebrovascular diseases : the official journal of National Stroke Association* 2014; **23**(5): 861-8.
5. Johnson BH, Bonafede MM, Watson C. Short- and longer-term health-care resource utilization and costs associated with acute ischemic stroke. *Clinicoecon Outcomes Res* 2016; **8**: 53-61.
6. Goeree R, Blackhouse G, Petrovic R, Salama S. Cost of stroke in Canada: a 1-year prospective study. *Journal of Medical Economics* 2005; **8**(1-4): 147-67.
7. Kim J, Andrew NE, Thrift AG, Bernhardt J, Lindley RI, Cadilhac DA. The potential health and economic impact of improving stroke care standards for Australia. *International Journal of Stroke* 2017; **12**(8): 875-85.
8. Stroke Foundation. 2020. The Economic Impact of Stroke in Australia 2020. Sydney: Stroke Foundation and Deloitte-Access Economics. https://strokefoundation.org.au/News/2020/11/04/02/57/No%20Postcode%20Untouched Accessed 12 August 2021.
9. Ng CS, Toh MP, Ng J, Ko Y. Direct medical cost of stroke in Singapore. *International journal of stroke : official journal of the International Stroke Society* 2015; **10 Suppl A100**: 75-82.
10. Chow WL, Tin AS, Meyyappan A. Factors Influencing Costs of Inpatient Ischaemic Stroke Care in Singapore. *Proceedings of Singapore Healthcare* 2010; **19**(4): 283-91.
11. Cha Y-J. The Economic Burden of Stroke Based on South Korea's National Health Insurance Claims Database. *International journal of health policy and management* 2018; **7**(10): 904-9.
12. Dewilde S, Annemans L, Peeters A, et al. Modified Rankin scale as a determinant of direct medical costs after stroke. *International journal of stroke : official journal of the International Stroke Society* 2017; **12**(4): 392-400.
13. Kolominsky-Rabas PL, Heuschmann PU, Marschall D, et al. Lifetime cost of ischemic stroke in Germany: results and national projections from a population-based stroke registry: the Erlangen Stroke Project. *Stroke* 2006; **37**(5): 1179-83.
14. Meretoja A, Kaste M, Roine RO, et al. Direct costs of patients with stroke can be continuously monitored on a national level: performance, effectiveness, and Costs of Treatment episodes in Stroke (PERFECT Stroke) Database in Finland. *Stroke* 2011; **42**(7): 2007-12.

15. Lorenzovici L, Székely A, Csanádi M, Gaál P. Cost Assessment of Inpatient Care Episodes of Stroke in Romania. *Front Public Health* 2020; **8**: 605919.
16. Strilciuc S, Grad DA, Mixich V, et al. Societal Cost of Ischemic Stroke in Romania: Results from a Retrospective County-Level Study. *Brain Sci* 2021; **11**(6).
17. Parody E, Pedraza S, García-Gil MM, Crespo C, Serena J, Dávalos A. Cost-Utility Analysis of Magnetic Resonance Imaging Management of Patients with Acute Ischemic Stroke in a Spanish Hospital. *Neurol Ther* 2015; **4**(1): 25-37.
18. Alvarez-Sabín J, Quintana M, Masjuan J, et al. Economic impact of patients admitted to stroke units in Spain. *Eur J Health Econ* 2017; **18**(4): 449-58.
19. Persson J, Ferraz-Nunes J, Karlberg I. Economic burden of stroke in a large county in Sweden. *BMC Health Serv Res* 2012; **12**: 341.
20. Lundström E, Smits A, Borg J, Terént A. Four-fold increase in direct costs of stroke survivors with spasticity compared with stroke survivors without spasticity: the first year after the event. *Stroke* 2010; **41**(2): 319-24.
21. van Eeden M, van Heugten C, van Mastrigt GAPG, van Mierlo M, Visser-Meily JMA, Evers SMAA. The burden of stroke in the Netherlands: estimating quality of life and costs for 1 year poststroke. *BMJ Open* 2015; **5**(11): e008220.
22. Mahler MP, Züger K, Kaspar K, et al. A cost analysis of the first year after stroke - early triage and inpatient rehabilitation may reduce long term costs. *Swiss Med Wkly* 2008; **138**(31-32): 459-65.
23. Chevreul K, Durand-Zaleski I, Gouépo A, Fery-Lemonnier E, Hommel M, Woimant F. Cost of stroke in France. *Eur J Neurol* 2013; **20**(7): 1094-100.
24. Gerzeli S, Tarricone R, Zolo P, Colangelo I, Busca MR, Gandolfo C. The economic burden of stroke in Italy. The EcLIPSE Study: Economic Longitudinal Incidence-based Project for Stroke Evaluation. *Neurol Sci* 2005; **26**(2): 72-80.
25. Patel A, Berdunov V, Quayyum Z, King D, Knapp M, Wittenberg R. Estimated societal costs of stroke in the UK based on a discrete event simulation. *Age and Ageing* 2019; **49**(2): 270-6.
26. Zhang H, Yin Y, Zhang C, Zhang D. Costs of hospitalization for stroke from two urban health insurance claims data in Guangzhou City, southern China. *BMC Health Services Research* 2019; **19**(1): 671.
27. Yin X, Huang L, Man X, et al. Inpatient Cost of Stroke in Beijing: A Descriptive Analysis. *Neuroepidemiology* 2018; **51**(3-4): 115-22.
28. Huo X, Jiang B, Chen Z, et al. Difference of hospital charges for stroke inpatients between hospitals with different levels and therapeutic modes in Beijing, China. *Int J Neurosci* 2017; **127**(9): 752-61.
29. Yong M, Xianjun X, Jinghu L, Yunyun F. Effect of health insurance on direct hospitalisation costs for in-patients with ischaemic stroke in China. *Aust Health Rev* 2018; **42**(1): 39-44.
30. Lu L, Pan J. The association of hospital competition with inpatient costs of stroke: Evidence from China. *Soc Sci Med* 2019; **230**: 234-45.
31. Wei JW, Heeley EL, Jan S, et al. Variations and determinants of hospital costs for acute stroke in China. *PloS one* 2010; **5**(9): e13041.
32. Zhu D, Shi X, Nicholas S, et al. Medical Service Utilization and Direct Medical Cost of Stroke in Urban China. *Int J Health Policy Manag* 2020.

33. Nor Azlin MN, Syed Aljunid SJ, Noor Azahz A, Amrizal MN, Saperi S. Direct medical cost of stroke: findings from a tertiary hospital in Malaysia. *Med J Malaysia* 2012; **67**(5): 473-7.
34. Vieira L, Safanelli J, Araujo T, et al. The cost of stroke in private hospitals in Brazil: a one-year prospective study. *Arq Neuropsiquiatr* 2019; **77**(6): 393-403.
35. Safanelli J, Vieira L, Araujo T, et al. The cost of stroke in a public hospital in Brazil: a one-year prospective study. *Arq Neuropsiquiatr* 2019; **77**(6): 404-11.
36. Christensen MC, Previgliano I, Capparelli FJ, Lerman D, Lee WC, Wainsztein NA. Acute treatment costs of intracerebral hemorrhage and ischemic stroke in Argentina. *Acta Neurol Scand* 2009; **119**(4): 246-53.
37. Asil T, Celik Y, Sut N, et al. Cost of acute ischemic and hemorrhagic stroke in Turkey. *Clin Neurol Neurosurg* 2011; **113**(2): 111-4.
38. Vo T, Tran H, Nguyen N, Nguyen H, Ha TV, Le NQ. Economic Aspects of Post-Stroke Rehabilitation: A Retrospective Data at a Traditional Medicine Hospital in Vietnam. *Journal of Clinical and Diagnostic Research* 2018.
39. Sribundit N, Riewpaiboon A, Chaikledkaew U, Stewart J, Tantirittisak T, Hanchaipiboolkul S. Cost of acute care for ischemic stroke in Thailand. *Southeast Asian Journal of Tropical Medicine and Public Health* 2017; **48**: 628-40.
40. Khealani BA, Javed ZF, Syed NA, Shafqat S, Wasay M. Cost of acute stroke care at a tertiary care hospital in Karachi, Pakistan. *J Pak Med Assoc* 2003; **53**(11): 552-5.
41. Diestro JDB, Omar AT, Sarmiento RJC, et al. Cost of hospitalization for stroke in a low-middle-income country: Findings from a public tertiary hospital in the Philippines. *International journal of stroke : official journal of the International Stroke Society* 2021; **16**(1): 39-42.
42. Viljoen C. Cost of acute stroke care in South Africa; 2014.
43. Kaur P, Kwatra G, Kaur R, Pandian JD. Cost of stroke in low and middle income countries: a systematic review. *International journal of stroke : official journal of the International Stroke Society* 2014; **9**(6): 678-82.
44. Maredza M, Chola L. Economic burden of stroke in a rural South African setting. *eNeurologicalSci* 2016; **3**: 26-32.
45. Aminde LN, Dzudie A, Mapoure YN, Tantchou JC, Veerman JL. Estimation and determinants of direct medical costs of ischaemic heart disease, stroke and hypertensive heart disease: evidence from two major hospitals in Cameroon. *BMC Health Serv Res* 2021; **21**(1): 140.
46. Kwatra G, Kaur P, Toor G, et al. Cost of stroke from a tertiary center in northwest India. *Neurology India* 2013; **61**(6): 627-32.
47. Bettger JP, Liu C, Gandhi DBC, Sylaja PN, Jayaram N, Pandian JD. Emerging Areas of Stroke Rehabilitation Research in Low- and Middle-Income Countries. *Stroke* 2019; **50**(11): 3307-13.
48. Brinda EM, Rajkumar AP, Enemark U, Attermann J, Jacob KS. Cost and burden of informal caregiving of dependent older people in a rural Indian community. *BMC Health Services Research* 2014; **14**(1): 207.
49. Pandian JD, Gandhi DB, Lindley RI, Bettger JP. Informal Caregiving: A Growing Need for Inclusion in Stroke Rehabilitation. *Stroke* 2016; **47**(12): 3057-62.
50. Barral M, Rabier H, Termoz A, et al. Patients' productivity losses and informal care costs related to ischemic stroke: a French population-based study. *European Journal of Neurology* 2021; **28**(2): 548-57.

51. Dewey HM, Thrift AG, Mihalopoulos C, et al. Informal care for stroke survivors: results from the North East Melbourne Stroke Incidence Study (NEMESIS). *Stroke* 2002; **33**(4): 1028-33.
52. Joo H, Dunet DO, Fang J, Wang G. Cost of informal caregiving associated with stroke among the elderly in the United States. *Neurology* 2014; **83**(20): 1831-7.
53. Skolarus LE, Freedman VA, Feng C, Wing JJ, Burke JF. Care Received by Elderly US Stroke Survivors May Be Underestimated. *Stroke* 2016; **47**(8): 2090-5.
54. Dewey HM, Sturm J, Donnan GA, et al. Incidence and outcome of subtypes of ischaemic stroke: initial results from the north East Melbourne stroke incidence study (NEMESIS). *Cerebrovascular Diseases* 2003; **15**(1-2): 133-9.
55. Gloede TD, Halbach SM, Thrift AG, Dewey HM, Pfaff H, Cadilhac DA. Long-term costs of stroke using 10-year longitudinal data from the North East Melbourne Stroke Incidence Study. *Stroke* 2014; **45**(11): 3389-94.
56. Meretoja A, Kaste M, Roine RO, et al. Direct Costs of Patients With Stroke Can Be Continuously Monitored on a National Level. *Stroke* 2011; **42**(7): 2007-12.
57. Bonner B, Pillai R, Sarma PS, Lipska KJ, Pandian J, Sylaja PN. Factors predictive of return to work after stroke in patients with mild-moderate disability in India. *European Journal of Neurology* 2016; **23**(3): 548-53.
58. van der Kemp J, Kruithof WJ, Nijboer TCW, van Bennekom CAM, van Heugten C, Visser-Meily JMA. Return to work after mild-to-moderate stroke: work satisfaction and predictive factors. *Neuropsychological Rehabilitation* 2019; **29**(4): 638-53.
59. Westerlind E, Persson HC, Sunnerhagen KS. Return to Work after a Stroke in Working Age Persons; A Six-Year Follow Up. *PLOS ONE* 2017; **12**(1): e0169759.
60. Peters GO, Buni SG, Oyeyemi AY, Hamzat TK. Determinants of return to work among Nigerian stroke survivors. *Disability and Rehabilitation* 2013; **35**(6): 455-9.
61. Maaijwee NAMM, Rutten-Jacobs LCA, Arntz RM, et al. Long-term increased risk of unemployment after young stroke: a long-term follow-up study. *Neurology* 2014; **83**(13): 1132-8.
62. Vyas MV, Hackam DG, Silver FL, Laporte A, Kapral MK. Lost Productivity in Stroke Survivors: An Econometrics Analysis. *Neuroepidemiology* 2016; **47**(3-4): 164-70.
63. Boushey H, Jane S, November G. There Are Significant Business Costs to Replacing Employees. 2012; 2012.
64. Gochhayat HB, Nanda NS. Attrition leads to employee turnover costs – a study of the Indian software industry. *Srusti Management Review* 2011; **4**: 47-52.
65. UN. Transforming our world: the 2030 agenda for sustainable development. New York, NY: United Nations. 2015.
66. Feigin VL, Forouzanfar MH, Krishnamurthi R, et al. Global and regional burden of stroke during 1990–2010: findings from the Global Burden of Disease Study 2010. *Lancet* 2014; **383**(9913): 245-54.
67. GBD 2019 Stroke Collaborators. Global, regional, and national burden of stroke and its risk factors, 1990–2019: a systematic analysis for the Global Burden of Disease Study 2019. *Lancet Neurology* 2021; **20**: 795-820 doi.org/10.1016/S474-4422(21)00252-0.
68. Hankey GJ. The global and regional burden of stroke. *The Lancet Global health* 2013; **1**(5): e239-40.
69. Arnett DK, Blumenthal RS, Albert MA, et al. 2019 ACC/AHA Guideline on the Primary Prevention of Cardiovascular Disease: Executive Summary: A Report of the American

College of Cardiology/American Heart Association Task Force on Clinical Practice Guidelines. *Circulation* 2019; **140**(11): e563-e95.

70. Owolabi MO, Akarolo-Anthony S, Akinyemi R, et al. The burden of stroke in Africa: a glance at the present and a glimpse into the future. *Cardiovascular journal of Africa* 2015; **26**(2 Suppl 1): S27-S38.
71. Feigin VL, Vos T, Nichols E, et al. The global burden of neurological disorders: translating evidence into policy. *The Lancet Neurology* 2020; **19**(3): 255-65.
72. Feigin VL, Brainin M, Norrving B, et al. What Is the Best Mix of Population-Wide and High-Risk Targeted Strategies of Primary Stroke and Cardiovascular Disease Prevention? *Journal of the American Heart Association* 2020; **9**(3): e014494.
73. Global Alliance for Chronic Diseases. About GACD. http://www.gacd.org/about Accessed on 7 July 2019.
74. Chobanian AV. Hypertension in 2017-what is the right target? *JAMA - Journal of the American Medical Association* 2017; **317**(6): 579-80.
75. Gorelick PB. Primary prevention of stroke: impact of healthy lifestyle. *Circulation* 2008; **118**(9): 904-6.
76. Tikk K, Sookthai D, Monni S, et al. Primary preventive potential for stroke by avoidance of major lifestyle risk factors: The European prospective investigation into cancer and nutrition-heidelberg cohort. *Stroke* 2014; **45**(7): 2041-6.
77. Rose G. Strategy of prevention: lessons from cardiovascular disease. *British medical journal (Clinical research ed)* 1981; **282**(6279): 1847-51.
78. Spence JD. Secondary stroke prevention. *Nat Rev Neurol* 2010; **6**(9): 477-86.
79. Feigin VL, Nguyen G, Cercy K, et al. The GBD 2016 Lifetime Risk of Stroke Collaborators. Global, Regional, and Country-Specific Lifetime Risks of Stroke, 1990 and 2016. *New Engl J Med* 2018; **379**(25): 2429-37.
80. Feigin VL, Norrving B, George MG, Foltz JL, Roth GA, Mensah GA. Prevention of stroke: a strategic global imperative. *Nature Reviews Neurology* 2016; **12**(9): 501-12.
81. Feigin VL, Roth GA, Naghavi M, et al. Global burden of stroke and risk factors in 188 countries, during 1990–2013: a systematic analysis for the Global Burden of Disease Study 2013. *The Lancet Neurology* 2016; **15**(9): 913-24.
82. Rose G, Khaw K-T, Marmot M. Rose's Strategy of Preventive Medicine: Oxford University Press; 2008.
83. Brainin M, Sliwa K. WSO and WHF joint position statement on population-wide prevention strategies. *The Lancet* 2020; **396**(10250): 533-4.
84. Brainin M, Feigin VL, Norrving B, Martins SCO, Hankey GJ, Hachinski V. Global prevention of stroke and dementia: the WSO Declaration. *The Lancet Neurology* 2020; **19**(6): 487-8.
85. Owolabi MO, Sarfo F, Akinyemi R, et al. Dominant modifiable risk factors for stroke in Ghana and Nigeria (SIREN): a case-control study. *The Lancet Global Health* 2018; **6**(4): e436-e46.
86. O'Donnell MJ, Chin SL, Rangarajan S, et al. Global and regional effects of potentially modifiable risk factors associated with acute stroke in 32 countries (INTERSTROKE): a case-control study. *Lancet* 2016; **388**(10046): 761-75.
87. Steiger N, Cifu AS. Primary prevention of stroke. *JAMA - Journal of the American Medical Association* 2016; **316**(6): 658-9.
88. Larsson SC, Åkesson A, Wolk A. Primary prevention of stroke by a healthy lifestyle in a high-risk group. *Neurology* 2015; **84**(22): 2224-8.

89. Johnson CO, Nguyen M, Roth GA, et al. Global, regional, and national burden of stroke, 1990–2016: a systematic analysis for the Global Burden of Disease Study 2016. *The Lancet Neurology* 2019; **18**(5): 439-58.
90. Sarfo FS, Ovbiagele B, Gebregziabher M, et al. Unraveling the risk factors for spontaneous intracerebral hemorrhage among West Africans. *Neurology* 2020; **94**(10): e998-e1012.
91. Statistica. Compound annual growth rate of global units and global mobile data traffic from 2015 to 2020, by device 2016. http://www.statista.com/statistics/276603/global-device-unit-and-mobile-data-traffic-cagr/.
92. Schoeppe S, Alley S, Van Lippevelde W, et al. Efficacy of interventions that use apps to improve diet, physical activity and sedentary behaviour: a systematic review. *The international journal of behavioral nutrition and physical activity* 2016; **13**(1): 127.
93. Zhao J, Freeman B, Li M. Can Mobile Phone Apps Influence People's Health Behavior Change? An Evidence Review. *Journal of medical Internet research* 2016; **18**(11): e287.
94. Rospo G VV, Bonomi AG, Thomassen IW, van Dantzig S, La Torre A, Sartor F. Cardiorespiratory Improvements Achieved by American College of Sports Medicine's Exercise Prescription Implemented on a Mobile App. *JMIR Mhealth Uhealth* 2016; **4**(2): e77.
95. Goode L. Tons of people are buying Fitbits, but are they actually using them? The Verge. 2015.
96. Cadmus-Bertram LA, Marcus BH, Patterson RE, Parker BA, Morey BL. Randomized Trial of a Fitbit-Based Physical Activity Intervention for Women. *American Journal of Preventive Medicine* 2015; **49**(3): 414-8.
97. Fritz T, Huang EM, Murphy GC, Zimmermann T. Persuasive technology in the real world: a study of long-term use of activity sensing devices for fitness. Proceedings of the SIGCHI Conference on Human Factors in Computing Systems. Toronto, Ontario, Canada: ACM; 2014. p. 487-96.
98. Wang Q, Egelandsdal B, Amdam GV, Almli VL, Oostindjer M. Diet and Physical Activity Apps: Perceived Effectiveness by App Users. *JMIR mHealth and uHealth* 2016; **4**(2): e33.
99. Jamaladin H, van de Belt TH, Luijpers LC, et al. Mobile Apps for Blood Pressure Monitoring: Systematic Search in App Stores and Content Analysis. *JMIR mHealth and uHealth* 2018; **6**(11): e187-e.
100. Masaki K, Tateno H, Nomura A, et al. A randomized controlled trial of a smoking cessation smartphone application with a carbon monoxide checker. *npj Digital Medicine* 2020; **3**(1): 35.
101. Lüscher J, Berli C, Schwaninger P, Scholz U. Smoking cessation with smartphone applications (SWAPP): study protocol for a randomized controlled trial. *BMC Public Health* 2019; **19**(1): 1400.
102. Feigin VL, Krishnamurthi R, Bhattacharjee R, et al. New Strategy to Reduce the Global Burden of Stroke. *Stroke* 2015; **46**(6): 1740-7.
103. Wolf PA, D'Agostino RB, Belanger AJ, Kannel WB. Probability of stroke: A risk profile from the Framingham Study. *Stroke* 1991; **22**(3): 312-8.
104. Parmar P, Krishnamurthi R, Ikram MA, et al. The Stroke Riskometer App: validation of a data collection tool and stroke risk predictor. *International Journal of Stroke* 2015; **10**(2): 231-44.

105. Meschia JF, Bushnell C, Boden-Albala B, et al. Guidelines for the primary prevention of stroke: A statement for healthcare professionals from the American Heart Association/American Stroke Association. *Stroke* 2014; **45**(12): 3754-832.
106. Krishnamurthi R, Bhattacharjee R, Parmar P, et al. Effectiveness of the Stroke Riskometer App for Primary Stroke Prevention: A Pilot RCT. Final Program of the International Stroke Conference in Los Angeles, USA 23-26 January 2018. *Late-Breaking News Science Abstracts of of the International Stroke Conference 24 January 2018* 2018; **Presentation Number LBP18**: page 85.
107. Krishnamurthi R, Barker-Colllo S, A. T, et al. Mobile technology for primary stroke prevention: a proof-of-concept pilot randomised controlled trial - a brief report. *Stroke* 2018; **50**: 196-8.
108. Goris J, Komaric N, Guandalini A, Francis D, Hawes E. Effectiveness of multicultural health workers in chronic disease prevention and self-management in culturally and linguistically diverse populations: A systematic literature review. *Australian Journal of Primary Health* 2013; **19**(1): 14-37.
109. Beratarrechea A, Lee AG, Willner JM, Jahangir E, Ciapponi A, Rubinstein A. The Impact of Mobile Health Interventions on Chronic Disease Outcomes in Developing Countries: A Systematic Review. *Telemedicine & e-Health* 2014; **20**(1): 75-82.
110. Feigin VL, Norrving B, Mensah GA. Primary prevention of cardiovascular disease through population-wide motivational strategies: insights from using smartphones in stroke prevention. *BMJ Global Health* 2017 Apr 4; **2**(2):e000306): DOI: 10.1136/bmjgh-2017-000306
111. Kaptoge S, Pennells L, De Bacquer D, et al. World Health Organization cardiovascular disease risk charts: revised models to estimate risk in 21 global regions. *The Lancet Global Health* 2019; **7**(10): e1332-e45.
112. Brainin M, Feigin VL, Norrving B, Martins S, Hachinski V, Hankey GJ. Декларация по глобальной первичной профилактике инсульта и деменции Всемирной организации по борьбе с инсультом. *Annals of Clinical and Experimental Neurology* 2020; **14**(3): 5-10.
113. Krishnamurthi R, George A, Merkin A, Bhattacharjee R, Feigin VL. Can we stop the stroke tsunami? Mitigating the barriers, amplifying the facilitators. *Journal of the Royal Society of New Zealand* 2020: 1-20.
114. Martins SC, Sacks C, Hacke W, et al. Priorities to reduce the burden of stroke in Latin American countries. *The Lancet Neurology* 2019; **18**(7): 674-83.
115. Brainin M, Feigin V, Bath PM, et al. Multi-level community interventions for primary stroke prevention: A conceptual approach by the World Stroke Organization. *International Journal of Stroke* 2019; **14**(8): 818-25.
116. Feigin VL. Anthology of stroke epidemiology in the 20th and 21st centuries: Assessing the past, the present, and envisioning the future. *International Journal of Stroke* 2019; **14(3)**: 223-37.
117. Bhattacharjee R, Hussain T. Digital data (mHealth and social media). In: Vasan RS, Sawyer D, eds. The Encyclopedia of cardiovascular research and medicine. Oxford, UK: Elsevier; 2018: 90-1.
118. Brainin M, Feigin V, Martins S, et al. Cut stroke in half: Polypill for primary prevention in stroke. *International Journal of Stroke* 2018; **13**(6): 633-47.
119. Feigin VL. Primary stroke prevention needs overhaul. *International Journal of Stroke* 2017; **12**(1): 5-6.

120. Béjot Y, Giroud M, Feigin VL. French version of the Stroke Riskometer™ App: A new tool to reduce the burden of stroke. *Revue Neurologique* 2017; **173**(4): 179.
121. Feigin VL, Varakin YY, Kravchenko MA, et al. A new approach to stroke prevention in Russia. *Human Physiology* 2016; **42**(8): 854-7.
122. Feigin VL, Krishnamurthi R. Stroke is largely preventable across the globe: where to next? *The Lancet* 2016.
123. Morgan J. The power of the App: can mobile-technology save lives? *The Lancet Neurology* 2015; **DOI 10.1016/S1474-4422(15)00297-5**.
124. Feigin VL, Wang W, Fu H, et al. Primary stroke prevention in China - a new approach. *Neurological research* 2015; **37**(5): 378-80.
125. Feigin VL, Krishnamurthi R, Bhattacharjee R. Putting your health in your own hands: A new strategy for primary stroke prevention. Brain Disorders & Therapy Global Summit on Stroke: Birmingham, UK; 2015: 88.
126. Bhattacharjee R, R. K, Hussain T, Purohit M. the Stroke Riskometer: A new tool to measure and monitor stroke risk. *International Journal of Stroke* 2015; **10**(S3): 52-84.
127. Norrving B, Davis SM, Feigin VL, Mensah GA, Sacco RL, Varghese C. Stroke Prevention Worldwide--What Could Make It Work? *Neuroepidemiology* 2015; **45**(3): 215-20.
128. Feigin VL, Norrving B, Wiebers DO. Approaches to Prevention of Cardiovascular Disease. *Jama* 2015; **314**(21): 2306-.
129. Feigin VL, Norrving B. A new paradigm for primary prevention strategy in people with elevated risk of stroke. *International Journal of Stroke* 2014; **9**(5): 624-6.
130. Wardlaw JM, Bath PM. Stroke research in 2018: extended time windows, refined benefit, and lifestyle prevention targets. *The Lancet Neurology* 2019; **18**(1): 2-3.
131. Hankey GL. Population Impact of Potentially Modifiable Risk Factors for Stroke. *Stroke* 2020; **51**(3): 719-28.
132. Brainin M, Sliwa K. WSO and WFN joint position statement on population-wide prevention strategies. *The Lancet* 2020; **396**: 533-4.
133. Parmar P, Krishnamurthi R, Ikram MA, et al. The Stroke Riskometer(TM) App: validation of a data collection tool and stroke risk predictor. *International Journal of Stroke: Official Journal of the International Stroke Society* 2015; **10**(2): 231-44.
